# Photonic Integrated Circuit Platform on 3 μm Thick Silicon‐on‐Insulator and Its Application Examples

**DOI:** 10.1002/nap2.70271

**Published:** 2026-09-02

**Authors:** Timo Aalto, Dura Shahwar, Markku Kapulainen, Mikko Harjanne, Katherine Bryant, Somnath Paul, Päivi Heimala, Fei Sun, Jae‐Wung Lee, Ari Hokkanen

**Affiliations:** ^1^ VTT Technical Research Centre of Finland Espoo Finland

**Keywords:** optical phased array, optical true time delay, photonic integrated circuit, polarization management, silicon photonics, silicon‐on‐insulator, thick SOI

## Abstract

This paper gives an overview of the 3 μm SOI PIC platform and explains its unique characteristics, such as ultra‐high mode confinement, low losses, dense integration, polarization‐independent operation, small phase errors, ultra‐broadband operation, and ability to handle relatively large optical powers. Examples of monolithically integrated passive and active waveguide components are given, including couplers, wavelength (de)multiplexers, phase modulators, and Ge photodiodes. Hybrid integration and packaging possibilities are briefly addressed. As examples of potential applications, some results are provided for tunable lasers, optical beam steering, medical imaging, and RF beam steering. Ongoing and targeted developments for this PIC platform are also briefly discussed, and a comparison to some alternative PIC platforms is made. Access to this technology in VTT’s Micronova cleanroom with 200 mm wafer size is supported by process design kits and the PIXEurope pilot line.

## Graphical abstract

This paper gives an overview of the 3 μm SOI PIC platform and explains its unique characteristics, such as ultra‐high mode confinement, low losses, dense integration, polarization‐independent operation, small phase errors, and ultra‐broadband operation. As examples of potential applications, some results are provided for tunable lasers, optical beam steering, medical imaging, and RF beam steering.

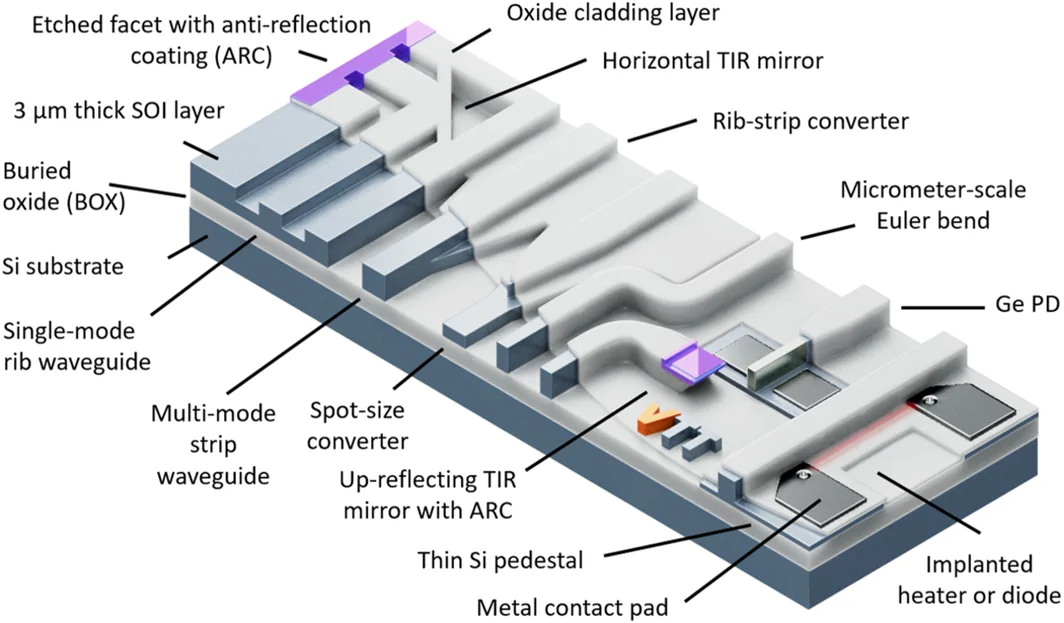

## 1 Introduction

Photonic integrated circuit (PIC) technologies are rapidly evolving and offer the possibility to replace bulky photonic systems with compact photonic chips. The number of potential application areas for PICs is almost infinite and can be compared to the extensive adoption of electronic integrated circuits in our society. The largest market for PICs to date has been in optical communication, especially inside data centers. The biggest driver for the development of PIC technology is now artificial intelligence (AI), which requires large number of optical transceivers to support the training and use of the AI models. However, PICs have the potential to be extensively used in numerous other applications with either large manufacturing volumes or otherwise significant impact. Promising application examples include optical spectroscopy, medical imaging, wearable sensors, light detection and ranging (LiDAR), RF beam steering in mobile networks, satellite communication, and photonic computing. There is currently no single PIC technology that could fulfill all application needs. This necessitates the development of complementary PIC technologies that together cover all relevant wavelength ranges and PIC functionalities (light sources, modulators, wavelength filters, detectors, etc.).

In this paper, we summarize the development status of the 3 μm thick‐silicon‐on‐insulator (SOI) waveguide PIC platform (Figure 1), and its unique aspects are described in detail. This includes the wavelength‐independent single‐mode operation of rib waveguides and maintaining fundamental mode light propagation even in multimode strip waveguides. Both rib and strip waveguides have ultra‐high mode confinement (Figure 2), which supports dense PIC integration, ultra‐broadband operation from 1.2 μm to over 3 μm wavelength, and ultra‐low propagation losses (< 3 dB/m). Recent demonstrations enabled by the ultra‐high mode confinement include ring resonators with intrinsic quality (*Q*) factors up to 14 million [1], polarization independent switching [2], stimulated Brillouin scattering in non‐suspended waveguides [3], and zero‐birefringence waveguides [4]. The feasibility of this PIC platform in quantum technologies was recently summarized [5].

**FIGURE 1:**
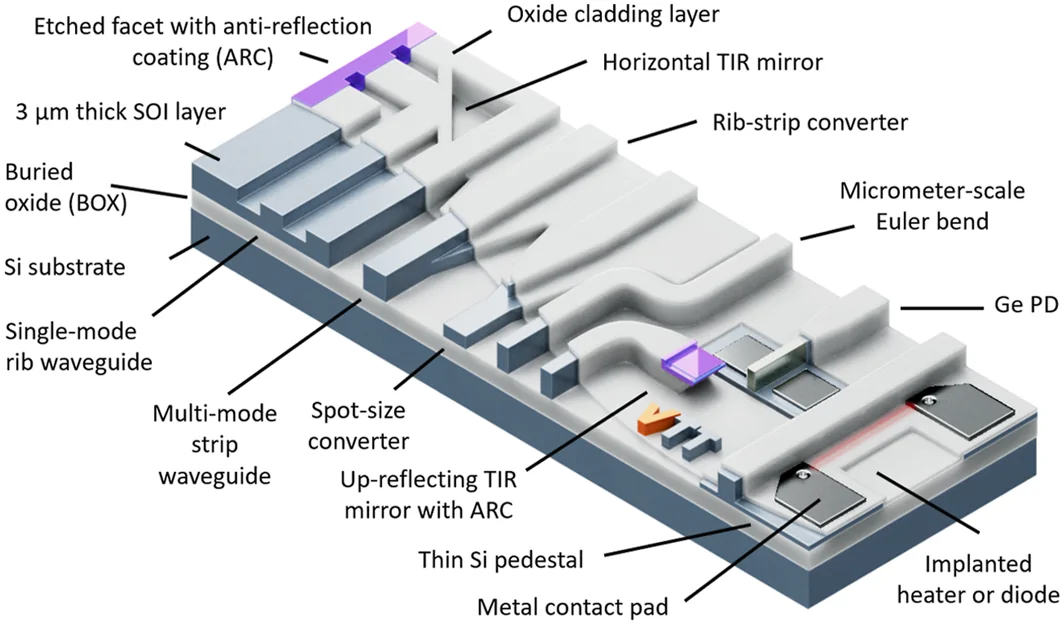
Schematic illustration of the 3 μm SOI PIC platform.

**FIGURE 2:**
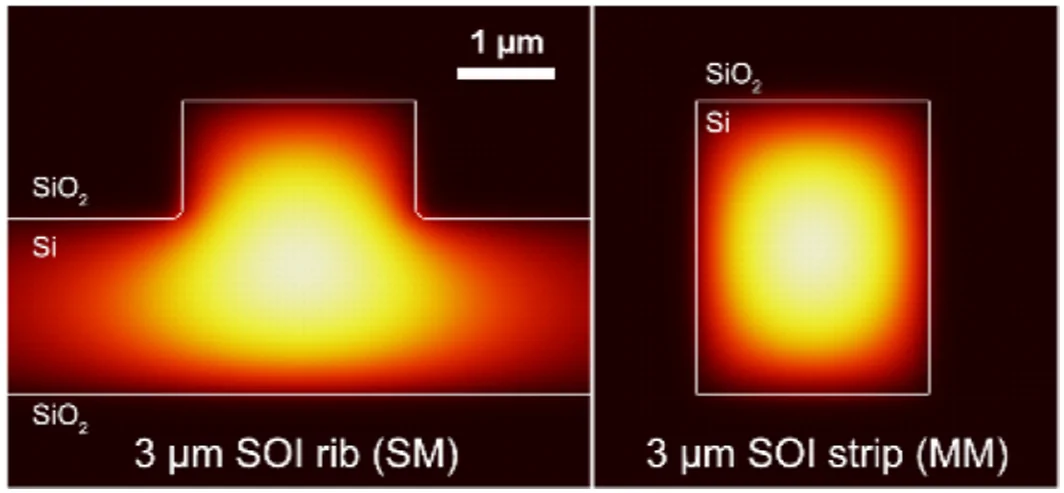
Simulated electric field profiles of the fundamental modes in 3 μm SOI rib and strip waveguides at 1550 nm wavelength (SM/MM = singe/multimode).

After explaining the basics of waveguides, crossings, bends, and mirrors in 3 μm SOI (in Section 2), we present illustrative results from polarization splitting and rotation, ultra‐broadband couplers and wavelength (de)multiplexers, modulators, photodetectors, flip‐chip integration, and PIC packaging (Sections 3, 4, 5, 6). The feasibility of the 3 μm SOI PIC platform is then demonstrated via application examples in Section 7: tunable lasers, optical beam steering with a passive optical phased array (OPA), medical ultrasound imaging, and RF beam steering with optical true time delay (OTTD) chips.

Based on the explained capabilities of the 3 μm SOI PIC platform, we compare it to some of the alternative PIC platforms in Section 8. Finally, the conclusions are given and the ongoing and targeted development of new capabilities on the PIC platform are briefly discussed in Section 9. Some of these developments are carried out in the PIXEurope pilot line [6], which also supports open access to this PIC platform and offers process design kits to facilitate the access.

## 2 Fundamental and Unique Properties of the 3 μm SOI Waveguides

### 2.1 Single‐Mode Rib and Multimode Strip Waveguides

Despite the large refractive index contrast (∼2) between the silicon core and the silicon dioxide cladding, the SOI rib waveguides offer single‐mode (SM) operation for a large variety of thicknesses, from < 1 to > 10 μm, and for an ultra‐wide wavelength range, from 1.2 to ∼6 μm, within silicon's transparency window. This unique property of SOI rib waveguides was recognized already in the early 1990s, which played a key role in initiating the development of silicon photonics [7]. If the rib height *h* is at least half of the waveguide thickness *H* and the waveguide width *W* obeys the equation

(1) $$W/H\leq 0.3+\left(h/H\right){\left[1-{\left(h/H\right)}^{2}\right]}^{-1/2},$$

the rib waveguide is single mode (Figure 3).

**FIGURE 3:**
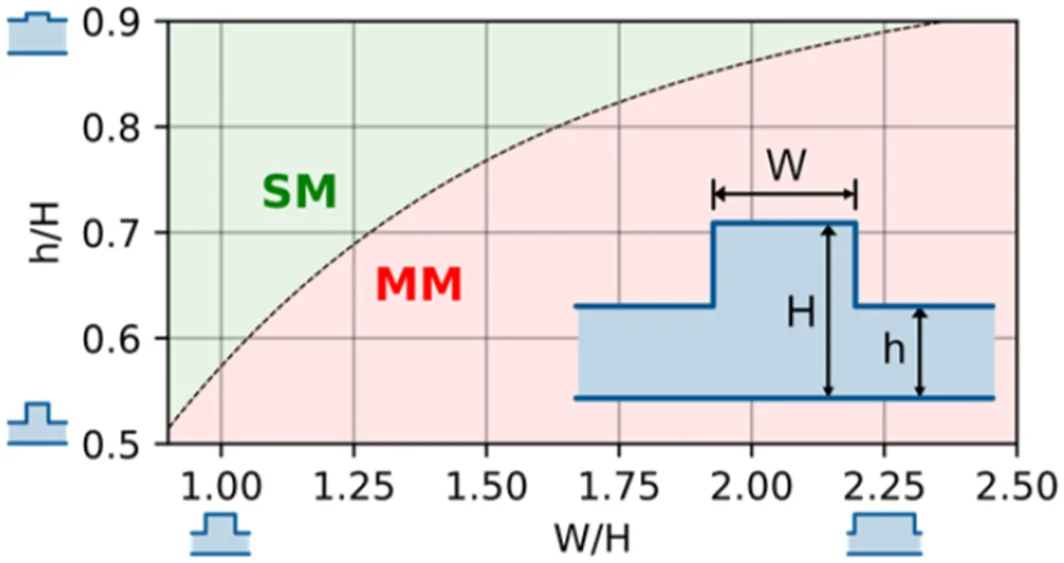
Single‐mode condition of the SOI rib waveguides (Equation 1) is independent of the absolute waveguide dimensions and wavelength; it only depends on the relative waveguide dimensions (*h*/*H* and *W*/*h*).

Strip waveguides, on the other hand, are strongly multimode (MM); unlike rib waveguides, strip waveguides support dense integration due to their high lateral mode confinement. The foundation for the 3 μm SOI PIC platform is the combined use of rib and strip waveguides and the ability to couple light adiabatically between them without exciting higher order modes [8]. This enables effectively SM and dense PICs, even when most of the circuit area is based on MM strip waveguides.

The fundamental mode is extremely well confined into the large silicon core in both rib and strip waveguides. As can be seen from Figure 4, the mode field is nearly identical for both polarizations and for very different wavelengths. This can also be concluded from Figure 5, which shows the dependence of the calculated effective index (*n*_eff_) on wavelength from 1.2 to 4 μm wavelength for both horizontal (TE) and vertical polarization (TM) in strip waveguides realized in 3 μm and 220 nm thick‐SOI strip waveguides. In 3 μm SOI, both the *n*_eff_ and the mode confinement have much smaller wavelength and polarization dependency compared to conventional waveguides. Effective index represents the weighted average of the refractive index in the materials where the waveguide mode propagates, so that the position of *n*_eff_ between the refractive indices of the core and cladding materials directly relates to the confinement of the mode in the core.

**FIGURE 4:**
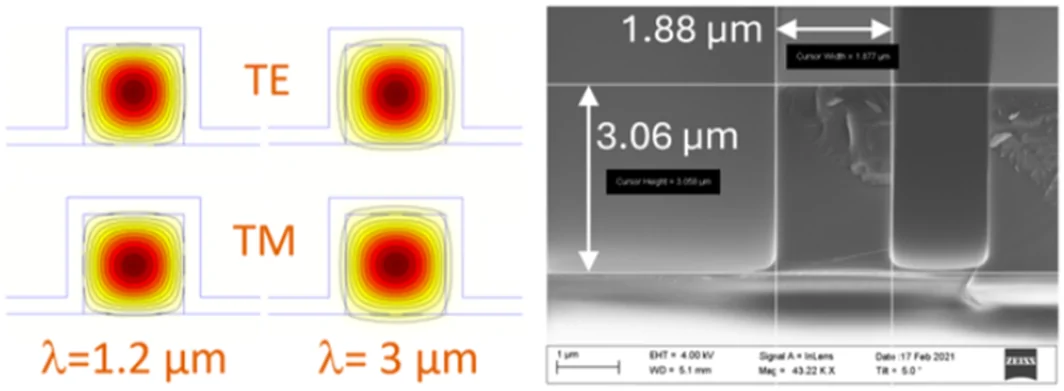
Left: Simulated mode profiles of 3 μm SOI strip waveguides at two polarizations and two wavelengths to illustrate ultra‐broadband and polarization‐independent operation. Right: Scanning electron microscope (SEM) image of a cleaved strip waveguide showing vertical and smooth sidewalls.

**FIGURE 5:**
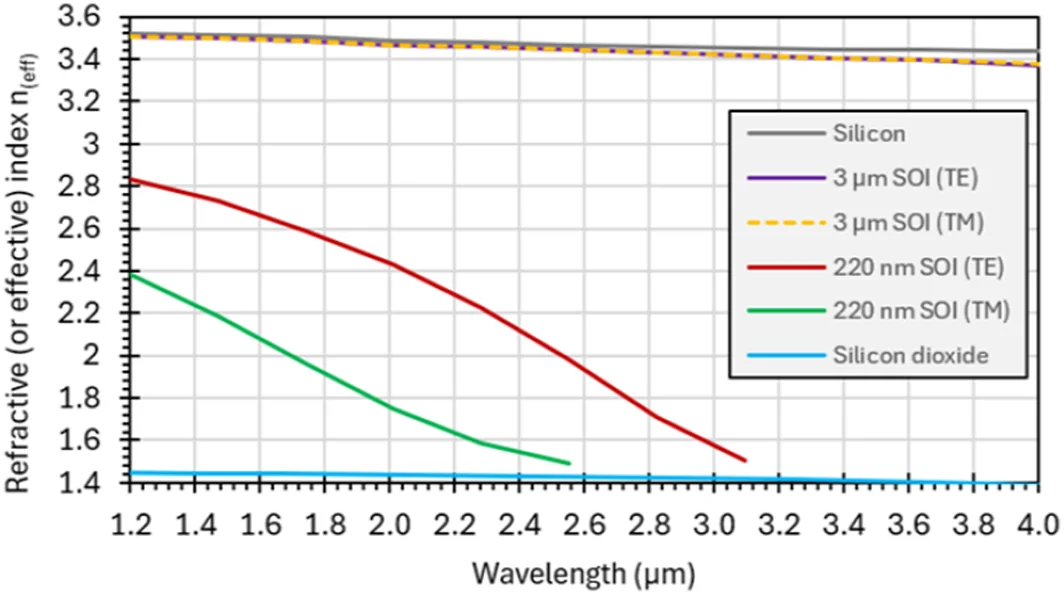
Simulated *n*_eff_ as a function of wavelength and polarization for strip waveguides in 3 μm and 220 nm thick SOI (widths 2.5 μm and 500 nm). The bulk refractive index of silicon and silicon dioxide from ref. [9] is shown in the same plot.

High confinement also enables very low losses (< 3 dB/m) [1], since the mode has very little overlap with the waveguide sidewall roughness. The propagation loss of the SOI waveguide is indeed almost exclusively due to sidewall roughness (at low optical power levels). By using hydrogen annealing for sidewall smoothing, the sidewall roughness can be significantly reduced (Figure 4), while keeping the sidewalls highly vertical. Furthermore, high mode confinement means the waveguide components are insensitive to small variations in the core dimensions, as the effective index very close to the refractive index of bulk silicon.

For wavelengths shorter than ∼2.2 μm, the maximum optical power in the SOI waveguides is limited by two‐photon absorption (TPA) and the resulting free carrier absorption (FCA). Those depend on the intensity of light. Due to larger core dimensions, the 3 μm SOI waveguides can tolerate optical powers up to ∼1 W, which is significantly higher than in submicron SOI waveguides. In practical implementations, the maximum optical power is often limited by packaging‐related factors such as facet quality, including antireflection coatings, and the power handling of spot‐size converters rather than the waveguide core itself. For example, polymer‐based micro‐optical lenses fabricated by two‐photon polymerization (see Section 6.2) can operate at multi‐watt continuous‐wave power levels, although their performance may still be influenced by absorption‐induced heating and thermal deformation [10]. Therefore, careful thermal and optical design of the packaging is essential to fully exploit the high‐power capability of thick‐SOI waveguides. In general, larger waveguide cross‐sections and mode fields lead to smaller intensities for a given optical power, making them more tolerant to damage or absorption caused by high optical power.

### 2.2 Waveguide Crossings, Bends, and TIR Mirrors

High mode confinement enables SOI waveguide crossings with minimal crosstalk and loss, and it facilitates horizontal or vertical reflection via total internal reflection (TIR) mirrors (Figures 1 and 6). This enables the realization of very dense PICs with low loss and low crosstalk. As can be seen from Figure 4, the fundamental optical modes for both TE and TM polarization have negligible amount of evanescent field in the oxide cladding surrounding the Si core. Thus, almost the entire mode hits the reflecting TIR mirror facet. For horizontal light reflection (Figure 6), the reflecting facet is formed from the sidewall of the waveguide and typically leads to an insertion loss (IL) of 0.1–0.2 dB per mirror [11]. This loss is caused by the finite roughness and tilt of the dry‐etched side‐wall, and by the horizontal divergence of light in the inner corner of the turning waveguide. Linewidth changes in waveguide processing can also cause losses, but typically these are negligible. In an up‐reflecting mirror (URM), the reflecting facet is wet‐etched to form a negative 45° facet, which is almost atomically smooth (Figure 1). The IL of the URM is more difficult to measure as light couples out of the waveguide and one cannot have a large cascade of URMs. Waveguide‐fiber coupling with an URM has been demonstrated with down to ∼0.5 dB coupling loss, which includes the impact of an anti‐reflection coating (ARC), waveguide‐fiber mode mismatch and the finite misalignment of the fiber. The IL of the URM also depends on the control of the wet‐etch parameters, which can affect the exact position and shape of the mirror facet.

Conventional waveguides and fibers enable SM operation by forcing higher order modes to radiate power out through the core‐cladding interface, which requires that the fundamental mode has a significant amount of its power carried by the evanescent field outside the core. For example, in 220 nm thick‐SOI waveguides, 15%–60% of the optical power typically propagates outside the Si core, depending on wavelength, polarization, and the waveguide width. This significantly reduces the efficiency of TIR‐based mirrors in conventional SM waveguides and in thin high‐index‐contrast MM waveguides, such as 220 nm SOI, that are locally widened to lower the propagation losses. Light traveling outside the core doesn’t get reflected, unless the mirror facet is somehow extended to the surrounding cladding, which is difficult to realize in practice. In 3 μm SOI, on the other hand, the amount of power traveling outside the core is typically less than or close to 0.1%, so that practically all light gets reflected. High mode confinement into the core also avoids the back‐reflection and scattering of light in waveguide crossings (Figure 6).

**FIGURE 6:**
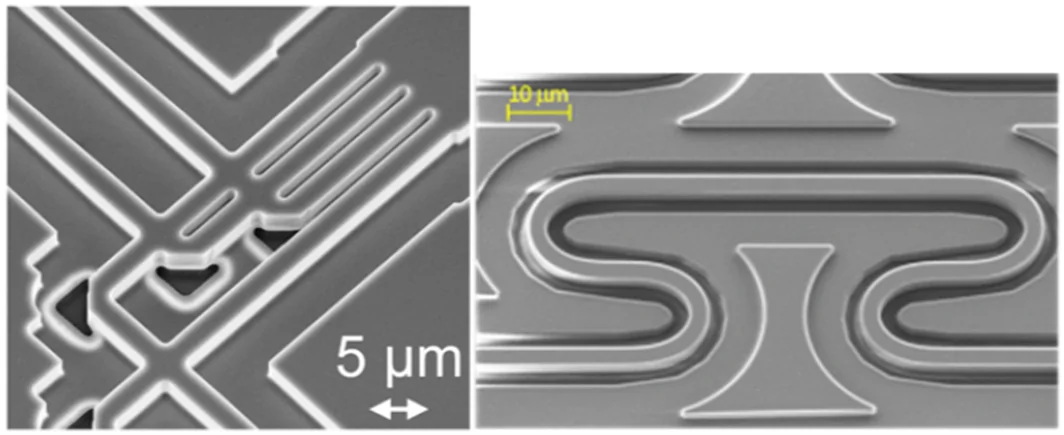
SEM images of fabricated PICs. Left: Waveguide crossings and TIR mirrors for dense waveguide routing. Right: Ultra‐compact Euler bends [12].

Compact Euler bends are a lossless alternative to TIR mirrors for turning the waveguides (Figure 6) [12]. With linearly varying curvature they can have minimum bending radius typically in the 10–50 μm range with bending losses below measurement limits. The specific shape of the Euler bend minimizes the excitation of light in the bent MM strip waveguides and allows to achieve 1–2 orders of magnitude smaller effective bend radii compared to conventional arc bends. The ultra‐high mode confinement of 3 μm SOI waveguides minimizes the outward radiation of power away from the fundamental waveguide mode. While this is counterintuitive, the thicker‐SOI waveguides can have even smaller bend radii than the thinner‐SOI waveguides, as long as the bending radius is limited by radiation losses. Thick‐SOI waveguides have been demonstrated to reach < 0.1 dB/90° bending loss with minimum bending radii down to 1 μm.

## 3 Polarization Management

### 3.1 Zero‐Birefringence Waveguides

One key benefit of the thick‐SOI platform compared to waveguides with submicron dimensions and high index‐contrast is the possibility for polarization‐independent operation in strip waveguides [4]. Bulky polarization diversity schemes can thus be avoided in 3 μm SOI. Some applications, like Faraday rotation and quantum networks, specifically require zero birefringence waveguides.

We have recently demonstrated 3 μm SOI strip waveguides with either zero group index birefringence (Δ*n*_g_) or zero effective index birefringence (Δ*n*_eff_), depending on the waveguide width.

The zero Δ*n*_eff_ waveguide width was identified using two different analysis methods, namely a set of arrayed waveguide gratings (AWG) and a novel measurement technique using folded waveguide structures (Figure 7a). The waveguide width required for achieving zero Δ*n*_eff_ at 1550 nm is ∼3.2 μm (Figure 8).

**FIGURE 7:**
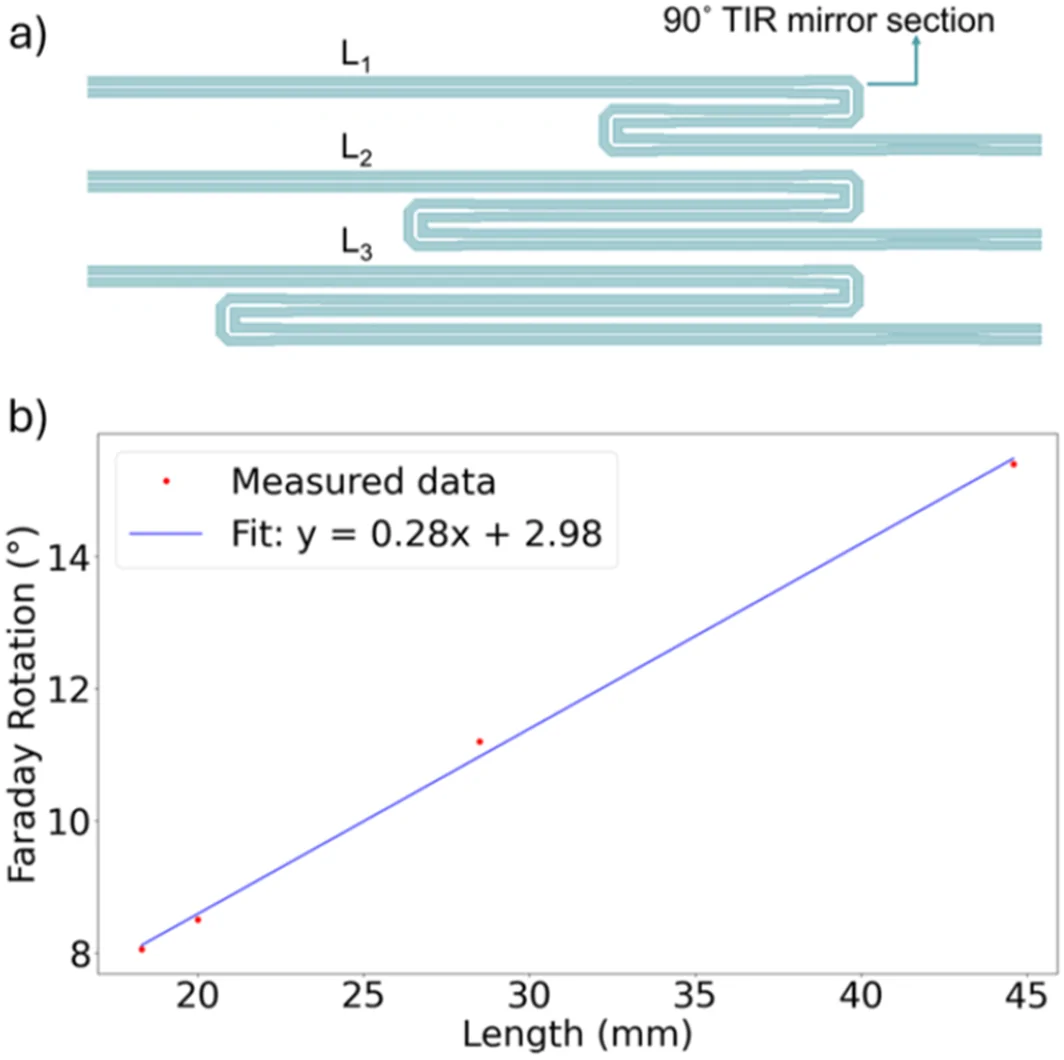
Faraday rotation in thick silicon waveguides: (a) Schematic of folded waveguide test structures [4]. (b) Measured and fitted Faraday rotation as a function of waveguide length.

**FIGURE 8:**
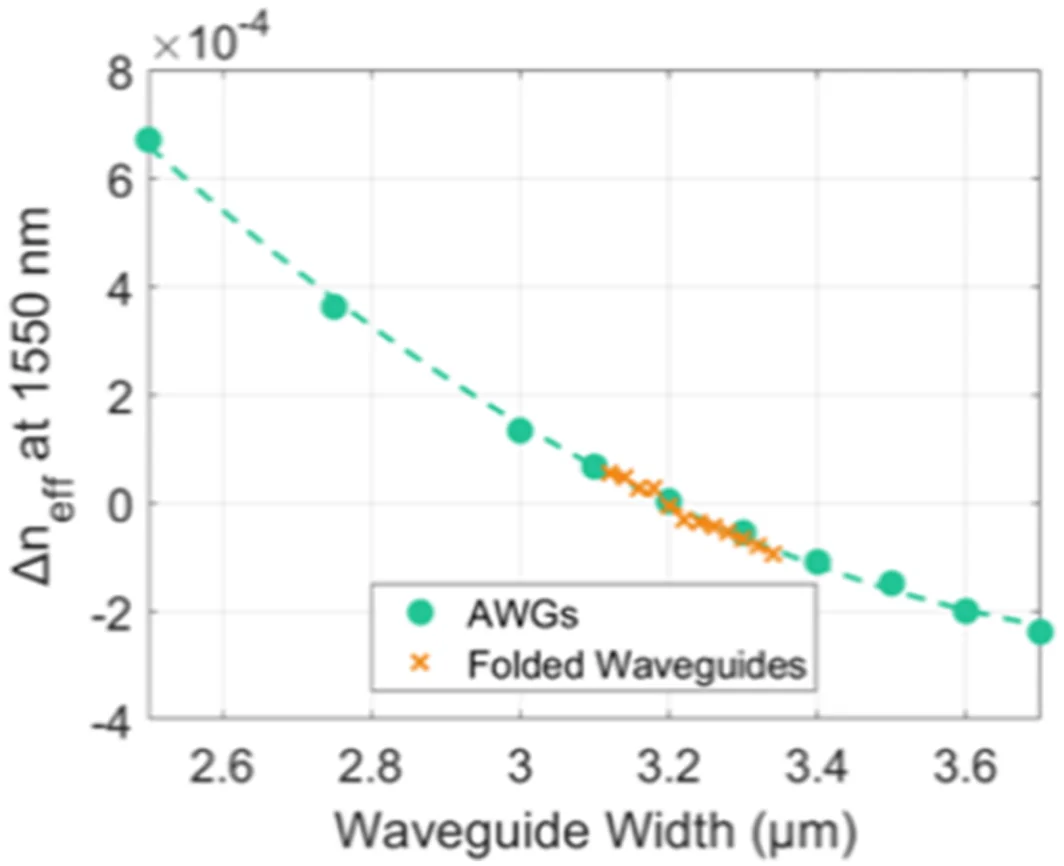
Measured effective index birefringence versus waveguide width in 3 μm thick‐SOI waveguides at 1550 nm. A zero Δ*n*_eff_ is achieved for a strip waveguide width of 3.2 μm [4].

The zero Δ*n*_g_ waveguide width was identified using three different analysis methods: the fixed analyzer method, the Fabry–Perot method, and via calculations from AWG data (using the relationship between group index, effective index, and dispersion). Zero Δ*n*_g_ is achieved for strip waveguides of width of ∼2.6 μm for 1550 nm (Figure 9).

**FIGURE 9:**
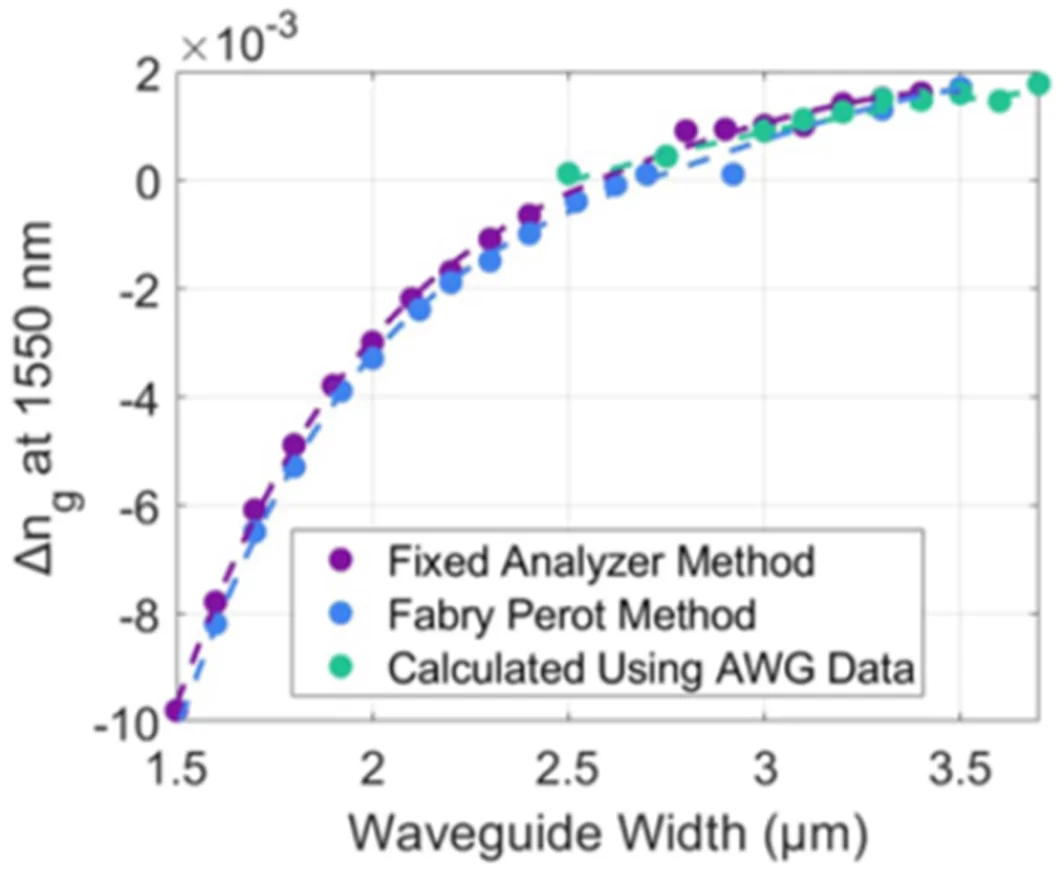
Measured group index birefringence versus waveguide width in 3 μm thick‐SOI waveguides at 1550 nm. A zero Δ*n*_g_ is achieved for a strip waveguide width of 2.6 μm [4].

The difference of waveguide width for achieving zero Δ*n*_eff_ and Δ*n*_g_ is due to the non‐zero waveguide dispersion. This naturally limits the ability to have fully polarization independent operation over a wide wavelength range. Depending on the targeted functionality, either Δ*n*_eff_ or Δ*n*_g_ can be minimized. In wavelength (de)multiplexing it is important to minimize the polarization‐dependent shifts of the spectra around the central wavelength, so that Δ*n*_eff_ should be primarily minimized. The non‐zero Δ*n*_g_ then limits the bandwidth for polarization‐independent multiplexing. Despite the dispersion of Δ*n*_eff_, it can be kept within ± 6 × 10^−6^ over 150 nm bandwidth at around 1550 nm wavelength, so that the polarization‐dependent peak shifts can be kept within ± 0.03 nm within this range. This is very low polarization dependency compared to many other PIC platforms, where peak shifts as high as 1.5 nm are sometimes claimed to represent “polarization insensitive” operation [13]. In Faraday rotation it is also important to minimize Δ*n*_eff_, while Δ*n*_g_ limits the bandwidth for polarization rotation. In some microwave photonics applications and nonlinear functions, it can be more important to keep the difference of two wavelengths polarization‐independent without necessarily aligning those wavelength pairs (TE and TM pairs). In such rare cases, one should minimize Δ*n*_g_, but in most cases one should minimize Δ*n*_eff_.

### 3.2 Polarization Splitters and Rotators

Micron‐scale waveguides can support both TE and TM polarization and have highly similar spatial mode distributions. As a result, these are considered as polarization‐independent waveguides. However, certain applications, such as telecommunications, sensing, and quantum key distribution, need polarization splitters and rotators [14]. Conversely, for applications such as isolators and circulators, the minimum and zero birefringence waveguides are advantageous.

Currently, we are developing a variety of polarization components for polarization management in our platform. This includes polarization splitters, reciprocal polarization rotators, and Faraday rotators. We have successfully demonstrated two types of Mach–Zehnder interferometer (MZI)‐based polarization splitters, utilizing: (1) form birefringence (Figure 10a) [15] and (2) a TIR mirrors (Figure 10b) [16]. The former uses a 50:50 multimode interference (MMI) coupler and a narrow waveguide which is highly birefringent in one arm of the MZI, thereby introducing a π phase difference between two arms for one of the polarizations. This device exhibits polarization extinction ratio (PER) > 15 dB for 35 nm bandwidth for both polarizations. The simulated and measured performance of this splitter is shown in Figure 11. The latter polarization splitter used the concept of polarization‐dependent and wavelength independent phase shifts occurring in TIR mirrors. Each arm of the MZI contains four TIR mirrors with identical physical angles; however, the mirror angles differ between the two arms, as shown schematically in Figure 10b. These devices achieved 15 dB PER for 80 nm bandwidth (where the bandwidth is limited by the MMI) as shown in Figure 12.

**FIGURE 10:**
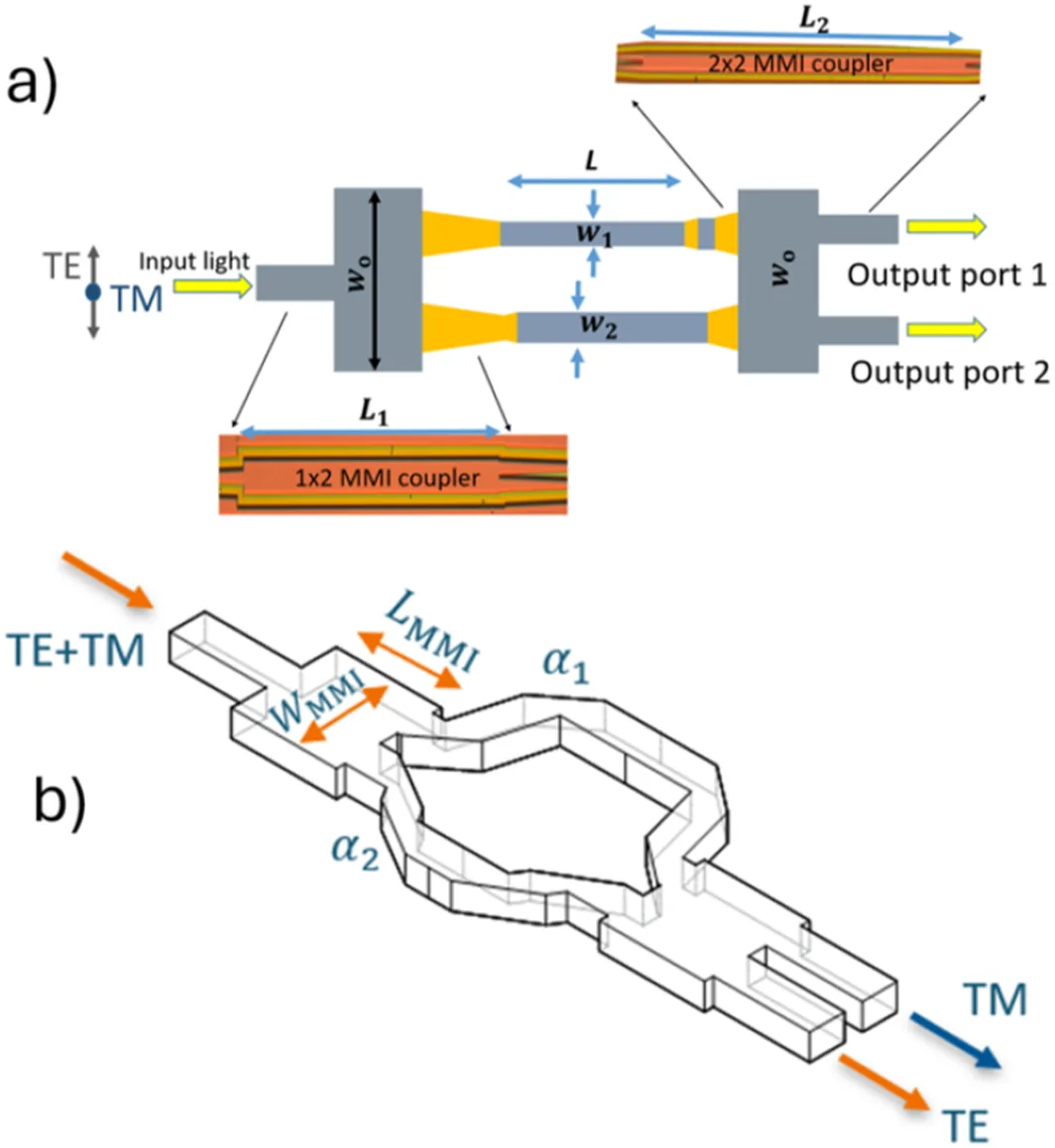
Schematic illustrations of on‐chip polarization splitters in thick SOI based on (a) form birefringence and (b) TIR mirrors.

**FIGURE 11:**
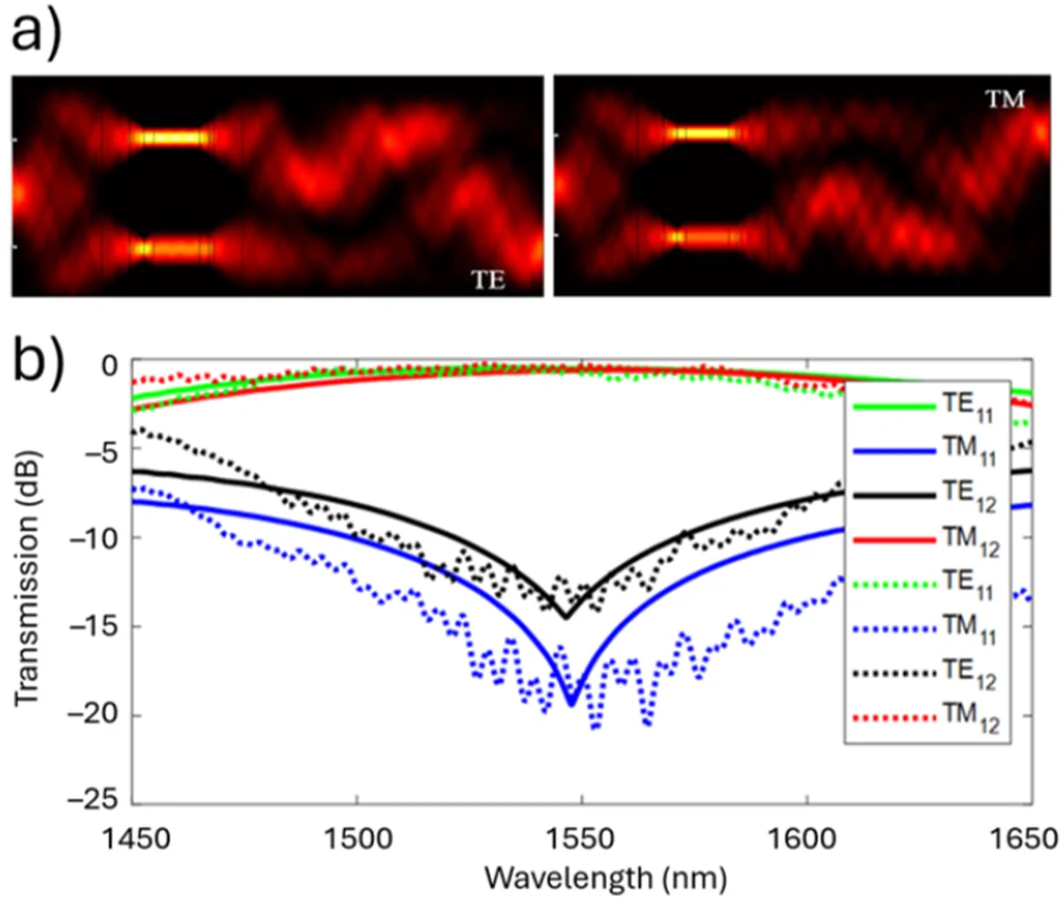
Birefringence‐based polarization splitter results: (a) Simulated wave propagation for TE and TM input polarization. (b) Analytical results (solid lines) compared with measurement results (dotted lines) [15].

**FIGURE 12:**
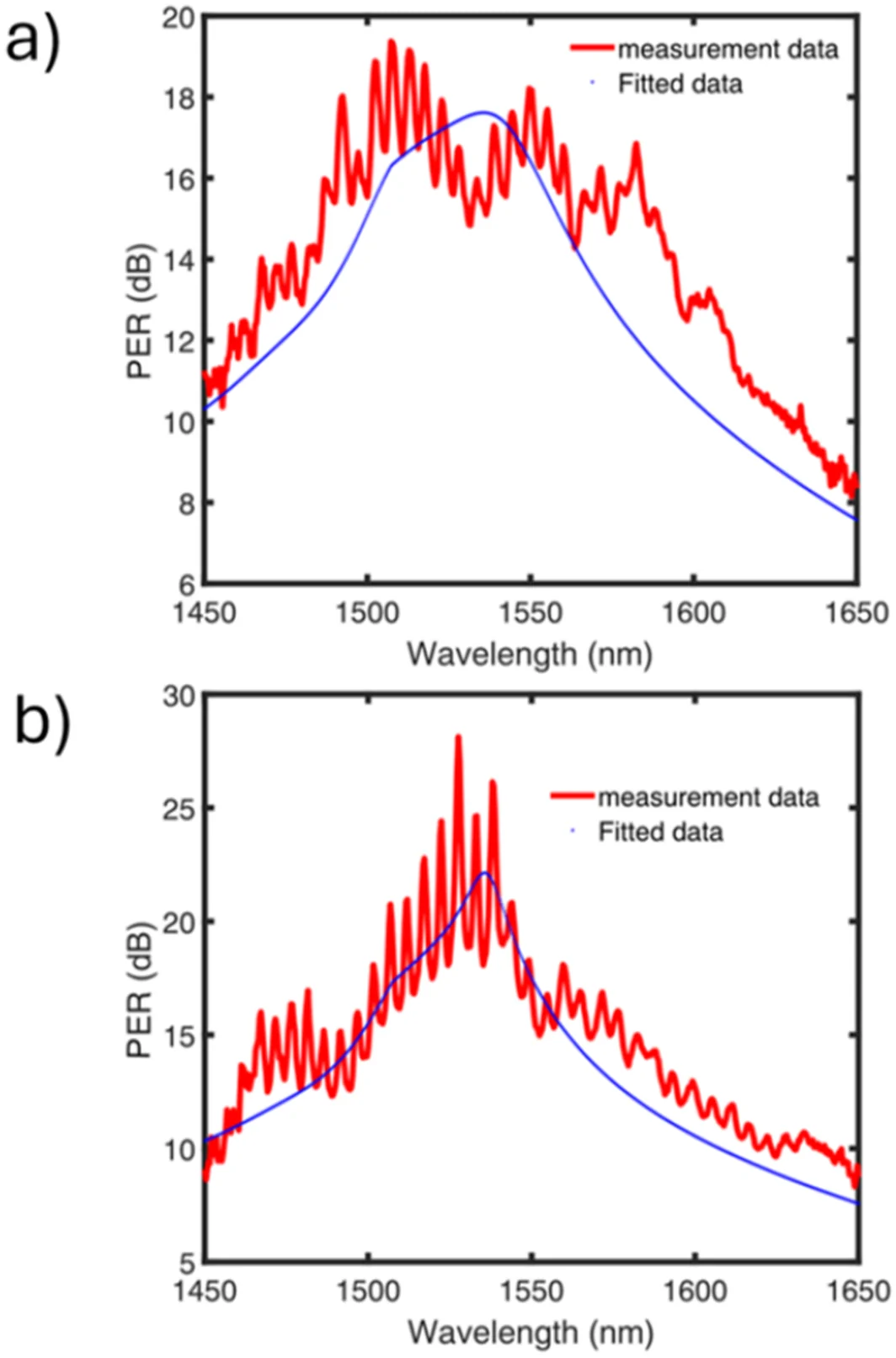
Polarization splitter results based on TIR mirrors as a function of wavelength for (a) TE and (b) TM input polarization. Measured data is shown with red line and fitted data with blue line [16].

For polarization rotation, we have demonstrated a nanostructured gold‐based metasurface waveplate device with high performance over a broad wavelength range (120 nm) with a polarization conversion efficiency (PCE) over 98.5% [17]. We also proposed and demonstrated an approach that provides a gateway to miniaturized optical devices enabling compact and broadband polarization rotation on a thick‐SOI platform. With the implemented metasurfaces, we have successfully demonstrated a compact on‐chip polarization rotator using nanorods on top of an up reflecting mirror (URM) on a thick‐SOI waveguide platform. The 3D schematic of our proposed metasurface and polarization rotation device and metasurface performance is shown in Figure 13.

**FIGURE 13:**
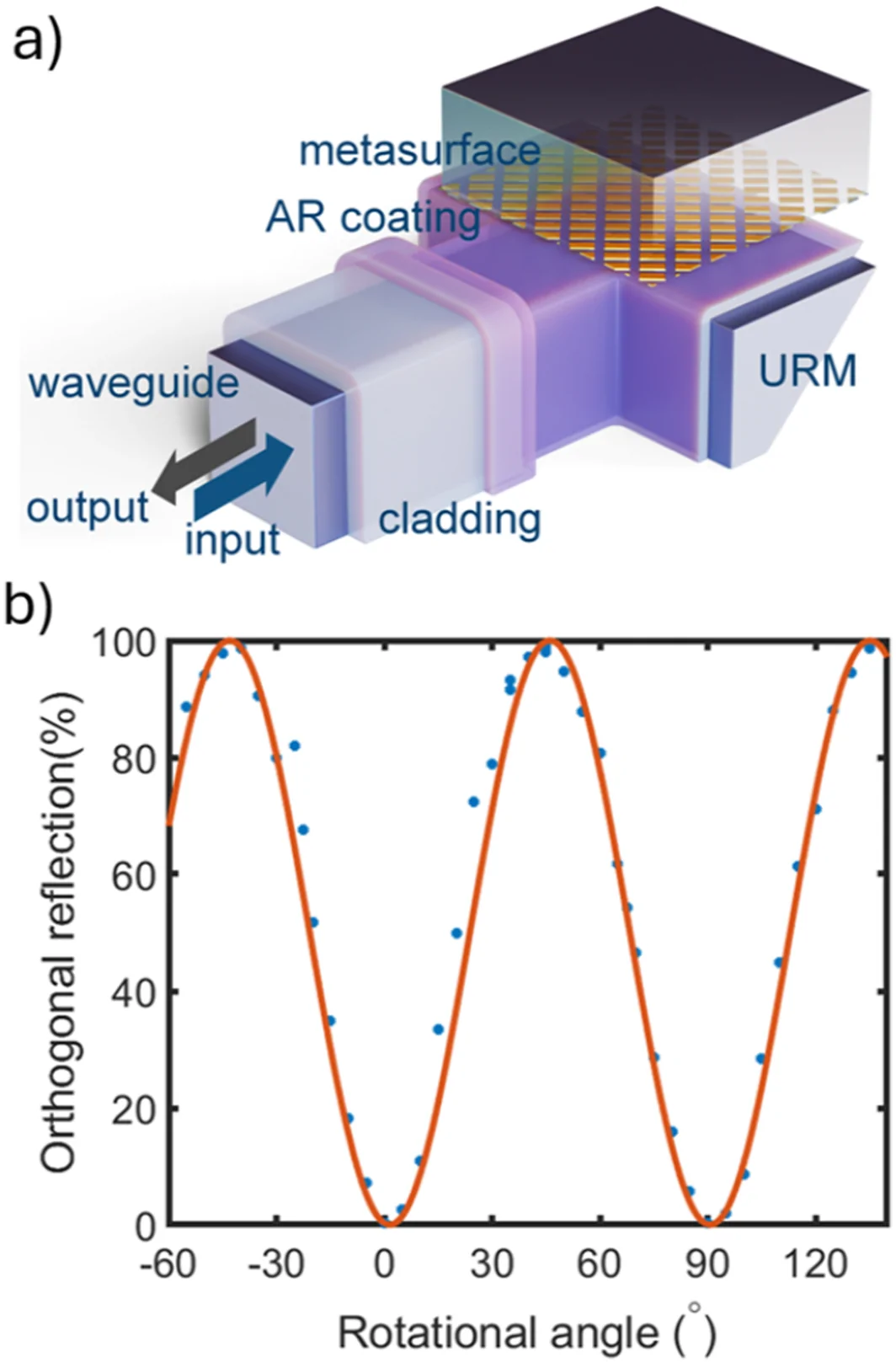
Polarization rotator on thick‐SOI platform [17]: (a) A three‐dimensional schematic for a metasurface‐based on‐chip polarization rotator. A 45° up‐reflecting mirror in the silicon waveguide is used to integrate with the metasurface on top. (b) Measured reflected power (presented with blue dots) for the polarization orthogonal to that of incident wave as a function of the rotational angle and a sinusoidal fit to the measured results (orange solid line).

Preliminary results of Faraday rotation are shown in Figure 7. We have used silicon itself as a magneto‐optic material in an externally applied magnetic field and achieved Faraday rotation in a zero‐birefringence waveguide [4]. TIR mirrors with straight waveguides were used to make folded waveguide test structure for Faraday rotation (and birefringence) measurements as shown in Figure 7a. Faraday rotation increases as the length of waveguide increases hence demonstrating first all‐silicon Fraday rotator, with achieved Faraday rotation 17°.

## 4 Ultra‐Broadband Passive Components

### 4.1 Ultra‐Broadband Couplers

Optical 1 × 2 and 2 × 2 couplers are some of the simplest and most versatile building blocks for PICs. They can be cascaded to form various interferometers, filters, and other more advanced functions. Y‐junctions and directional couplers (DC) represent the most traditional concepts for realizing 1 × 2 and 2 × 2 couplers, respectively. In general, 2 × 2 couplers are more versatile, as they allow to realize, for example, 2 × 2 MZIs and they can be optionally used as 1 × 2 couplers by simply leaving one of the four ports unused. But they are also more difficult to design, since it is more difficult to achieve the targeted coupling ratio (e.g., 50:50) to the two outputs when light cannot propagate symmetrically from one input to both outputs.

There are many alternatives for realizing 1 × 2 and 2 × 2 couplers in PICs. A Y‐junction with narrow splitting gap and small opening angle offers intrinsically symmetric 50:50 coupling with small wavelength dependency. It is possible to use 1 × 2 MMI couplers or inverse‐designed Y‐junction layouts to relax the requirement for a narrow splitting gap or to shorten the device [18]. Asymmetric Y‐junctions in 220 nm thick‐SOI slot waveguides have been demonstrated to offer 390 nm bandwidth for both polarizations with −3 + 0.5 dB transmission [19]. However, we primarily focus here on the more challenging 2 × 2 couplers.

MMI couplers (both 1 × 2 and 2 × 2) are short, easy to design and their power transmission spectra have a flat‐top shape. However, they have intrinsic wavelength dependency that typically limits their bandwidth to about 100 nm at around 1550 nm wavelength. With sub‐wavelength gratings their 1 dB bandwidth has been extended up to 300 nm [20]. Directional couplers tend to be much longer than MMI couplers, and their cross and bar‐port transmissions have sin^2^(*x*) and cos^2^(*x*) ‐shaped spectra, where *x* is inversely dependent on wavelength. The ideal operating point for a 50:50 DC has *x* = π/2, where the transmission changes rapidly as a function of wavelength and typically leads to narrow bandwidth.

In this paper, we present two 2 × 2 couplers that have been realized in 3 μm SOI waveguides with exceptionally broadband operation and without any sub‐micrometer dimensions, such as narrow gaps or dense gratings. The first one is a compact 2 × 2 (50:50) rib waveguide‐based polarization‐independent adiabatic coupler. Its design is similar to the one published earlier in 4 μm thick‐SOI waveguides [21], but optimized here for 3 μm SOI. The coupler has a remarkable bandwidth of 500 nm with measured cross and bar transmissions within −3 ± 0.5 dB (Figure 14). Further details about this coupler will be provided in a dedicated publication later.

**FIGURE 14:**
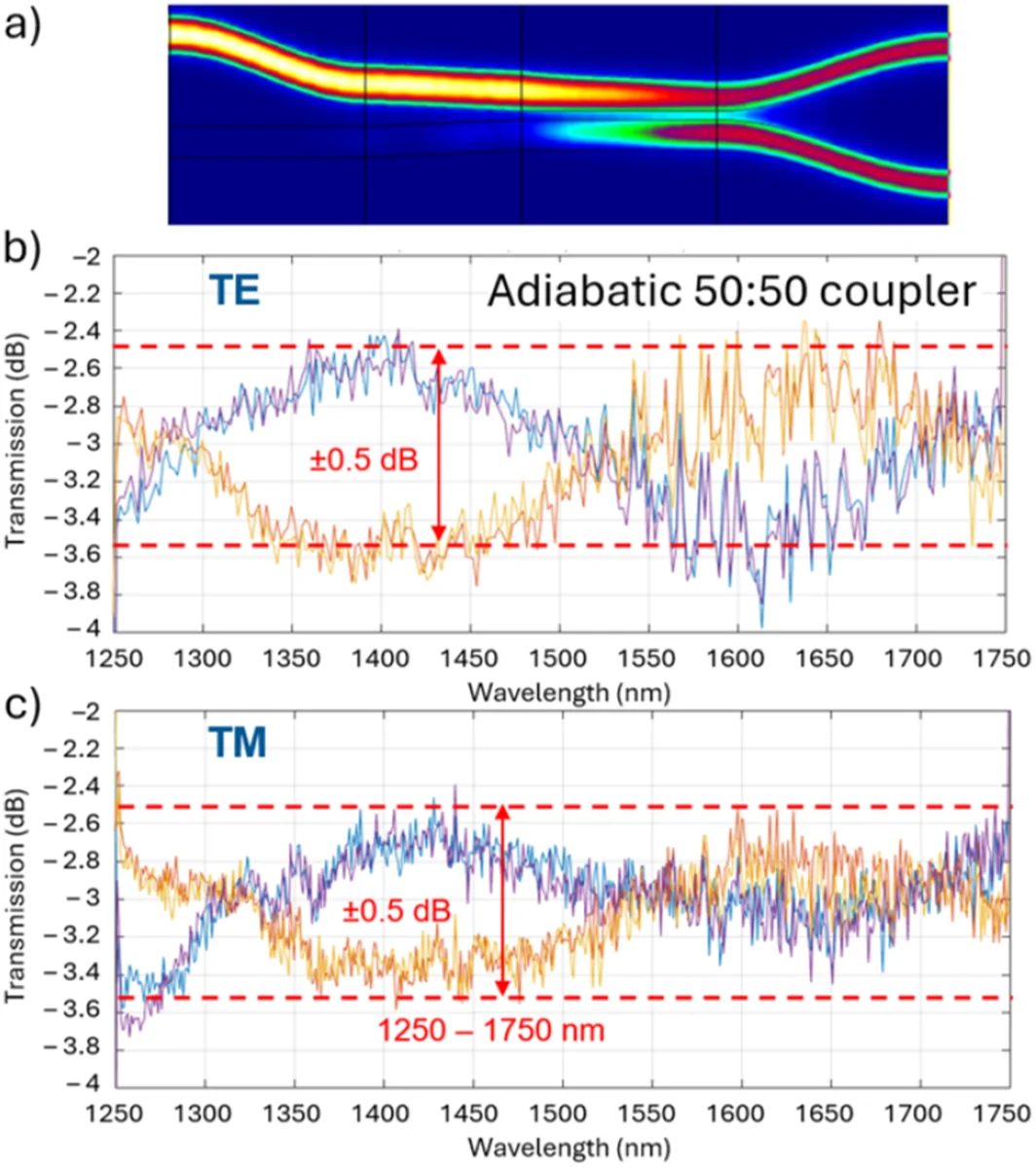
(a) Simulated and (b, c) measured power transmissions of the designed adiabatic 2 × 2 coupler. Measured deviation from the targeted 50:50 coupling (−3 dB transmission) is within ± 0.5 dB over a 500 nm bandwidth for both (b) TE and (c) TM polarization.

The second example result is an asymmetric directional coupler (aDC) that can act as a broadband 2 × 2 coupler for any target coupling ratio. A rib waveguide design is used, and light evanescently couples from the input waveguide to the output waveguide; the maximum coupling is the target coupling ratio, since the widths of the rib waveguides in the crossover region are different. By changing the widths of the two rib waveguides, the maximum coupling ratio can be designed to be anything between 0% and 100%. Here, we present results for a 2 × 2 aDC that was designed to have 14:86 dB power coupling ratio and targeted to be used as part of a tunable laser PIC. A top‐view schematic layout of the design and the simulated power splitting can be seen in Figure 15a,b. A schematic cross‐section of the asymmetric waveguide pair with two different waveguide widths is shown in the inset of Figure 15c. Measured cross and bar transmission spectra for the aDC are shown in Figure 15 with TE polarization. Targeted bar and cross transmissions are −0.7 and −8.6 dB, respectively. Measured bar and cross transmissions for both input ports vary within −1 ± 0.5 dB and −7.8 ± 0.5 dB from 1500 to 2250 nm, that is, with 750 nm bandwidth. The spectra remain fairly flat even up to 2400 nm, where the measurement noise becomes large due to the used super continuum light source. The result shows that the aDC components are significantly more broadband than conventional (symmetric) DCs or MMIs.

**FIGURE 15:**
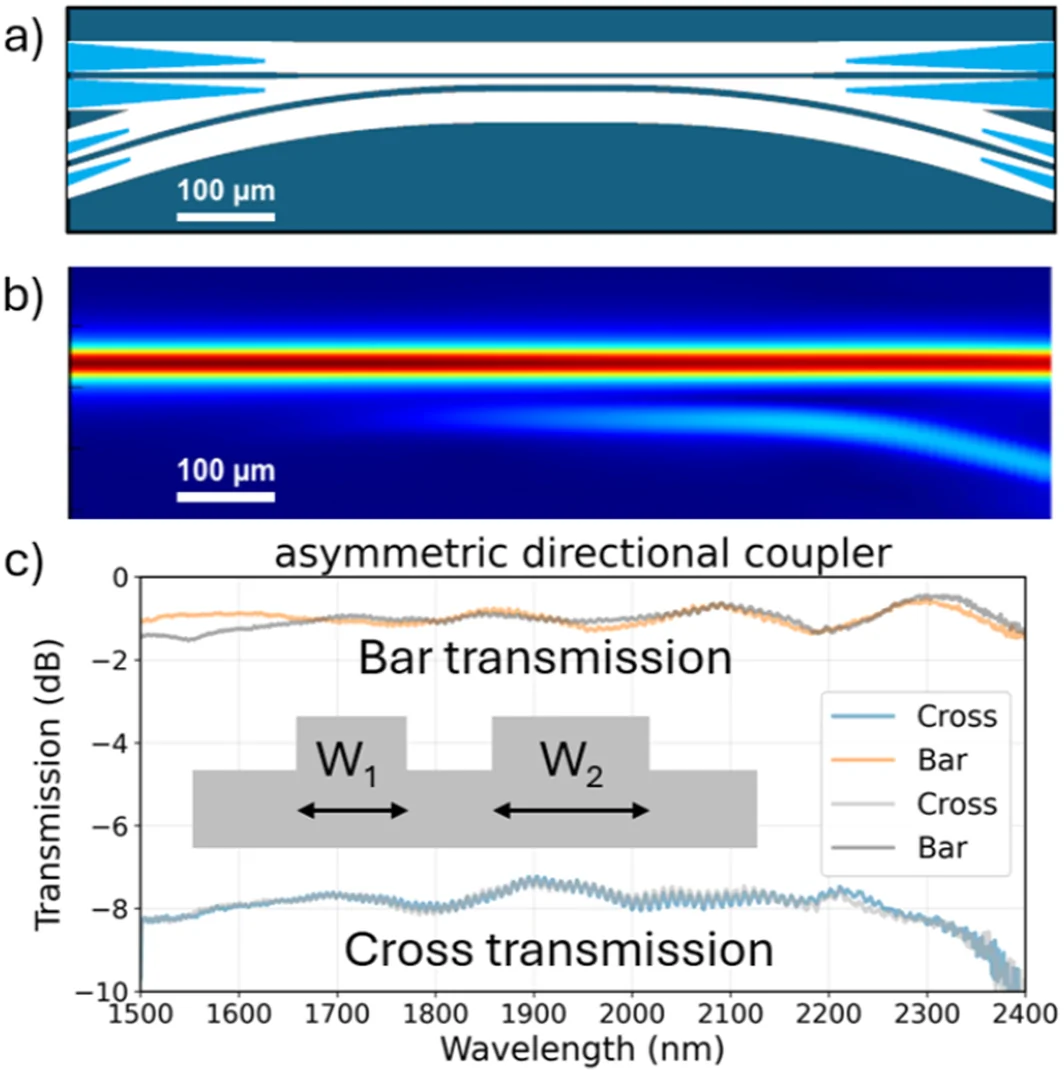
(a) Schematic top‐view of the aDC surrounded by rib‐strip converters. (b) Simulated transmission through the aDC. (c) Measured power transmissions (for TE polarization) from each input port to both output ports versus wavelength in an aDC design that targeted 14% cross coupling (−8.6 dB). Inset shows a schematic cross‐section of the aDC.

The reason for the exceptionally broadband operation is visualized in the analytically calculated graphs of Figure 16. Those show how operation around the maximum coupling point allows to stretch the bandwidth to almost 1200 nm, while keeping the imbalance of the coupler within ± 0.5 dB. In practice, coupling ratio depends on the waveguide dimensions and their variation due to fabrication tolerances. Large variations in the waveguide thickness, etch depth, or width can naturally influence the coupling. We expect that the maximum coupling ratio is most sensitive to the difference of the two waveguide widths (*W*_1_ and *W*_2_), which is set by the designer and very insensitive to fabrication imperfections. On the other hand, the length that is needed to achieve maximum coupling is sensitive to all waveguide dimensions and fabrication imperfections. Fortunately, the ultra‐broad bandwidth of the aDC makes it quite robust against changes in the optimum length of the coupler.

**FIGURE 16:**
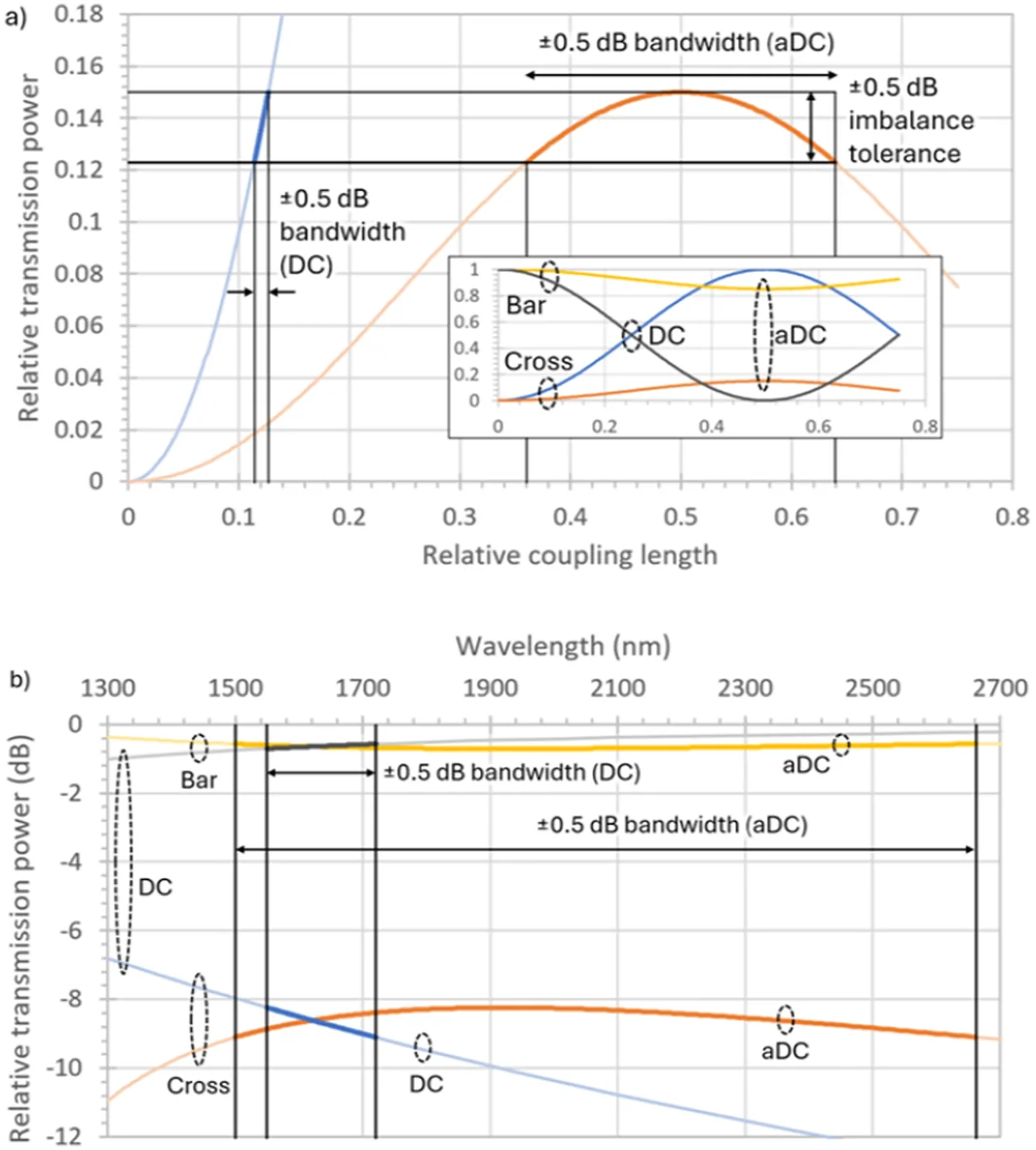
Illustration of the ability of an asymmetric directional coupler to offer wider bandwidth for a given imbalance tolerance when compared to a conventional symmetric DC. These simplified graphs have been analytically calculated without numerically calculating any optical mode fields or taking into account any losses or dispersion. (a) Relative power transmission to the cross‐port as a function of the relative coupler length. Inset shows the full transmission scale for bar and cross‐transmissions. (b) Relative power transmission (in dB units) to the cross‐port as a function of wavelength.

### 4.2 Ultra‐Broadband Wavelength Multiplexers

Wavelength (de)multiplexers (MUX) are fundamental components in integrated optics and are widely used in the C‐band and O‐band for telecommunication applications. The thick‐SOI waveguide technology offers the potential to extend MUX functionality beyond the telecom wavelengths and even into the mid‐infrared (MIR) region. In this regime, multiplexers can enable sensor applications, such as gas detection [22]. High‐resolution spectrometers based on multiplexers can be developed to resolve individual absorption lines of gases.

We have successfully demonstrated ultra‐broadband operation of SOI‐based multiplexers by characterizing arrayed waveguide gratings and echelle gratings at wavelengths beyond 2 μm. The results have been previously published [23], but we discuss and explain the results in more detail here. The measured components were originally designed for the 1.55 μm wavelength region and are based on 3 μm thick SOI waveguides.

The 1 × 12 AWG with 100 GHz channel spacing at 1.55 μm was measured in the 2.04 and 3.53 μm wavelength ranges. The measured transmission spectra are shown in Figure 17. The extinction ratio was approximately 20 dB, limited by the higher noise level of the measurement setup at longer wavelengths. At 1.55 μm, an extinction ratio of 30 dB was measured.

**FIGURE 17:**
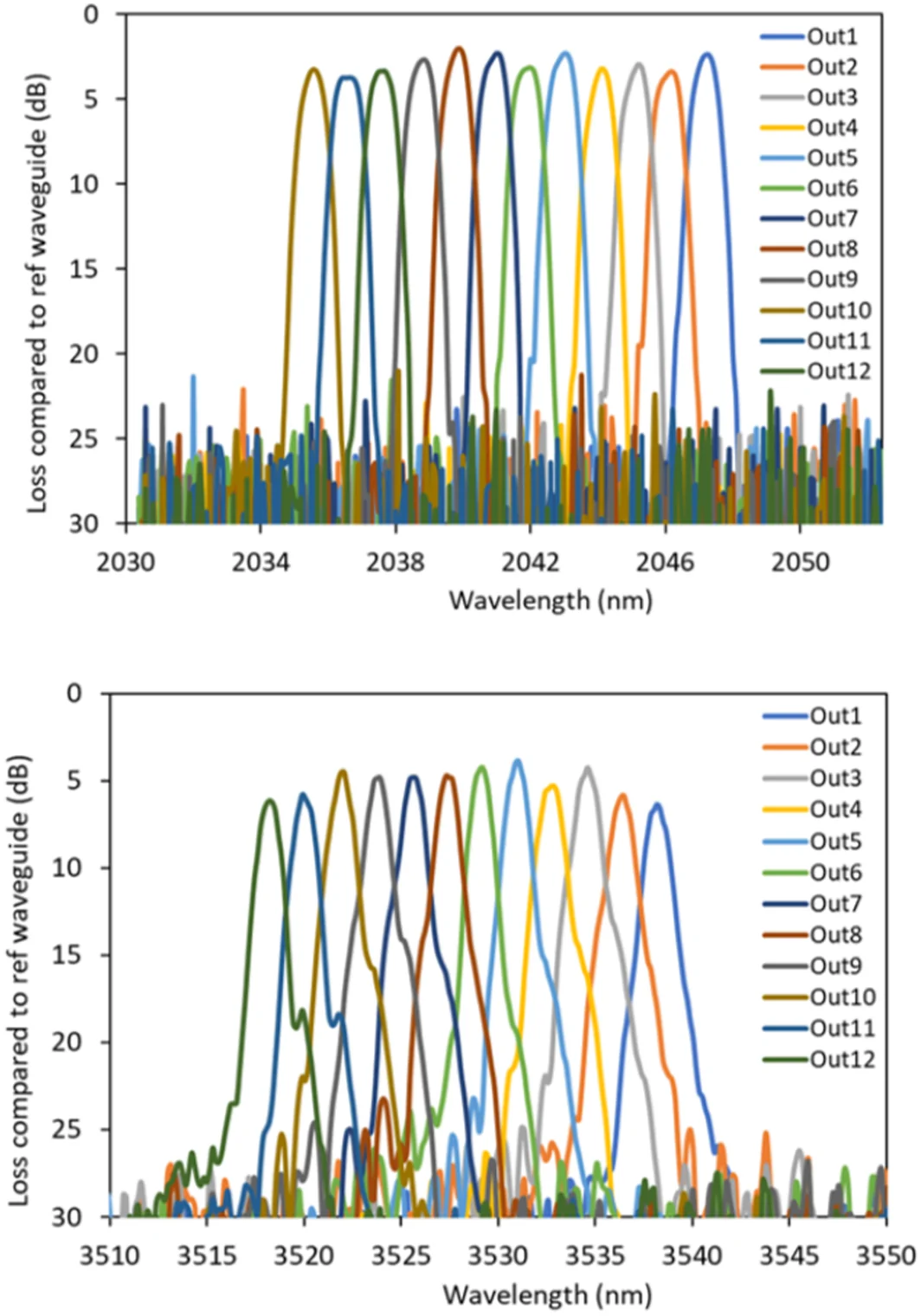
Measured transmission spectra of the 1 × 12 AWG for the 2.04 μm (top) and 3.53 μm (bottom) wavelength ranges [21].

The measured broadband transmission spectrum of the 1 × 5 echelle grating with 800 GHz channel spacing is presented in Figure 18. Across the measurement bandwidth, insertion loss was 3–4 dB, indicating stable performance over a 1100 nm wavelength span. The extinction ratio was consistently above 25 dB up to 2.1 μm. At wavelengths beyond 2.1 μm, the extinction ratio gradually decreased due to an increase in noise, which resulted from the limited output power of the supercontinuum light source used in the measurements.

**FIGURE 18:**
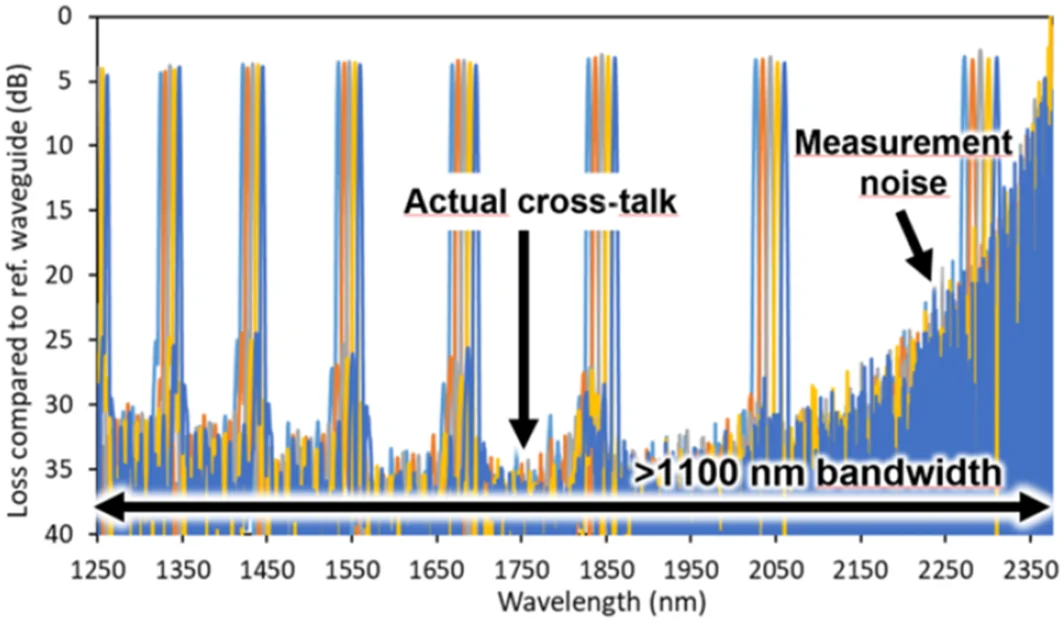
Measured transmission spectrum of the 1 × 5 echelle grating. The noise level increases after 2.1 μm wavelength due to the limited power of the used super continuum light source [23].

The underlying reason for the extremely broadband operation of the two multiplexers is the ultra‐high mode confinement in 3 μm SOI waveguides, as discussed above. The multiplexing function itself is based on the length differences of multiple optical paths either in strip waveguides (in AWG) or in the slab waveguide (in echelle grating). The effective index of both strip and slab waveguides in 3 μm SOI changes very slowly as a function of wavelength, as shown in Figure 5. This feature allows to keep the spacing of the wavelength channels fairly constant over an ultra‐wide spectral range, while the high confinement also allows to realize low‐loss bends in the waveguide array of the AWG.

Neither AWG nor echelle grating design involves MMI couplers or other interferometric components that would cause unwanted wavelength dependency beyond the targeted multiplexing function. The PICs that contain the multiplexers use Euler bends for turning light and rib‐strip converters to combine the dense integration of strip waveguides with the ultra‐broadband single‐mode operation of the rib waveguides. Conventional waveguides cannot offer such a combination of dense integration, low losses, and single‐mode operation over a wide wavelength range. Thus, the ultra‐broadband operation is enabled by the multiplexer itself and the rest of the waveguide circuit around it.

## 5 Modulators and Detectors

### 5.1 Thermo‐Optic Phase Modulators

Thermo‐optic (TO) phase shifters are widely used tuning elements in silicon photonics owing to their simple fabrication and control electronics. They utilize silicon's large thermo‐optic coefficient, which allows phase tuning even with modest temperature changes. The main drawback of TO phase shifters is their low switching speed, which is limited to well below MHz range. In thick SOI, heating is achieved with an implanted resistor located next to the optical waveguide on a thin silicon pedestal (see Figures 1 and 19). This geometry allows efficient heat transfer to the waveguide core without introducing optical losses from the implantation. The power required for π phase shift (*P*_π_) is around 25 mW, limited by the heat transfer to the Si substrate. The measured rise and fall times are below 15 µs. The length of the heater defines the device resistance, which for a typical 100 μm long device is around 1 kΩ. The length does not have a significant effect on the power required for π phase shift. Power efficiency of the phase shifters can be improved by reducing thermal conduction from waveguide to the environment. For this, we have demonstrated TO phase shifters fabricated on cavity SOI wafers from Okmetic [24]. The wafers had cavities etched on the Si substrate, matching the locations of the heaters, prior to wafer bonding (Figure 19). The improved thermal insulation reduced power consumption to *P*_π_ = 1.7 mW, but this comes with a penalty in increased rise and fall time of 200 µs.

**FIGURE 19:**
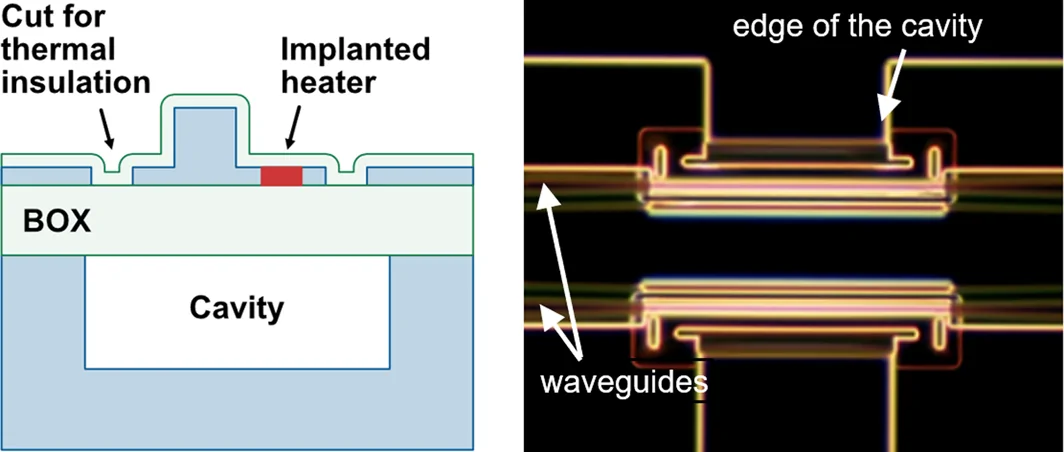
Left: A schematic cross‐sectional image of the 3 μm SOI waveguide and the implanted heater. Right: A dark field microscope image of two TO modulation arms in a fabricated MZI after waveguide formation. The edge of the cavity is seen as a thin red line.

### 5.2 Plasma Dispersion Modulators

Faster modulators can be realized by the free‐carrier plasma dispersion effect. Figure 20 schematically shows one example of an MZI‐based modulator, where one modulation arm is equipped with a phase shifter. One configuration of the phase shifter in our PIN modulator cross‐section is schematically shown in Figure 20. The PIN‐diode is formed from P‐ and N‐doped regions on a thin pedestal of the strip waveguide and the intrinsic region between them. Due to the plasma dispersion effect of Si, the refractive index of Si will decrease when free carriers (holes and electrons) are injected into the intrinsic regions from P and N regions.

**FIGURE 20:**
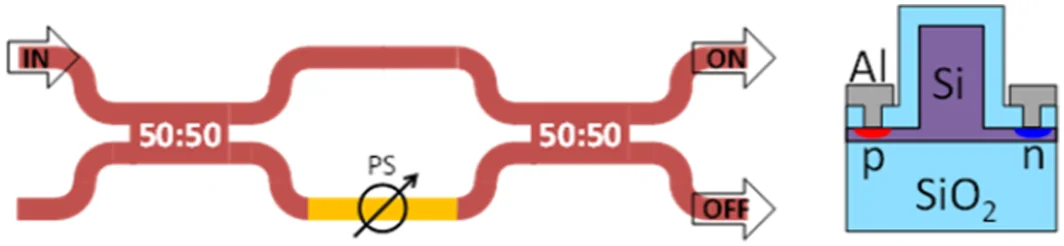
Schematic illustrations of MZI‐based modulator (left) and cross‐section of the modulation arm in a PIN electro‐optic modulator (right).

Since the movement of free carriers is much faster than thermal diffusion, PIN modulators show higher speed compared with their TO counterparts. The optical output of a PIN modulator driven by a square‐wave is shown in Figure 21. This modulator has a switching speed of 30 MHz, which represents an improvement of roughly three orders of magnitude when compared to TO phase shifters.

**FIGURE 21:**
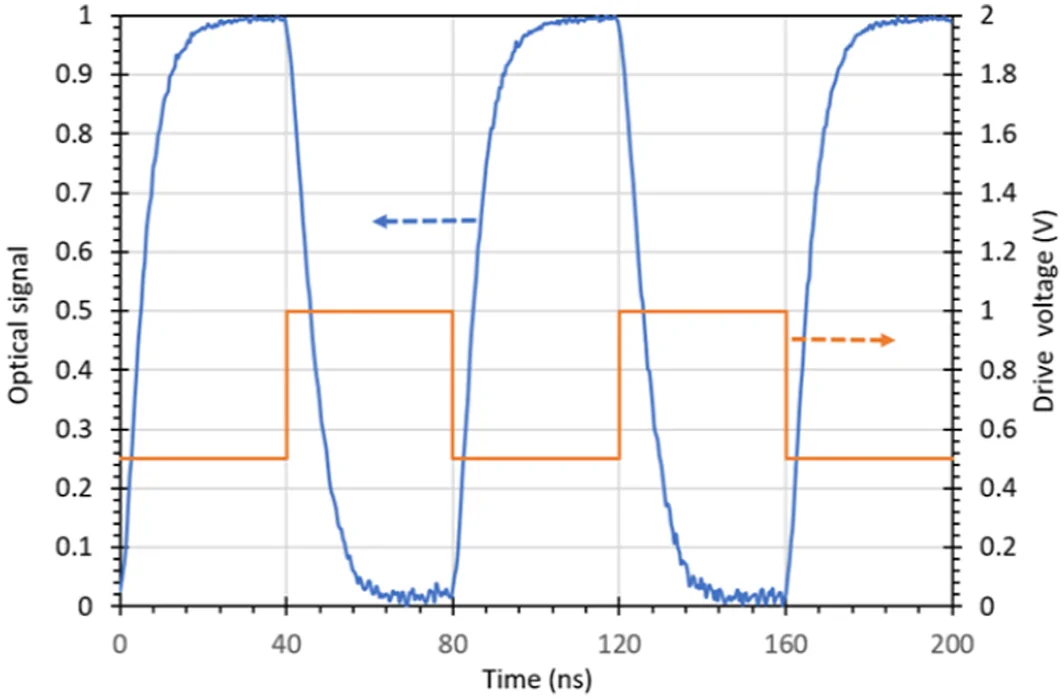
Measured optical output of a PIN modulator with square‐wave bias applied.

While thick‐SOI PICs offer many advantages, plasma dispersion modulators in 3 μm SOI are significantly slower than their thin‐SOI counterparts. In 220 nm thin SOI, the optical mode is confined to a small cross‐section, so the carrier‐depletion region overlaps a significant fraction of the mode, enabling efficient carrier depletion modulation [25]. In thick SOI, the large mode area and the difficulty in creating a sharp PN‐junction throughout the 3 μm thick core makes carrier depletion modulators less effective and the main focus has been on carrier injection modulators, which are inherently slower. Furthermore, the modulation bandwidth in both types of modulators is limited by the carrier transit times, and in larger waveguides, these are naturally much longer than in thin SOI. Thus, the lack of fast modulators is the biggest limitation in the 3 μm SOI PIC platform.

Modulator bandwidth could be improved through recombination lifetime reduction via ion implantation [26]. Another possibility is the hybrid or heterogeneous integration of materials with a stronger Pockels effect onto silicon, such as thin‐film lithium niobate (TFLN) [27] or barium titanate (BaTiO_3_) [28].

### 5.3 Ge Photodiodes

The Ge photodiode (PD) is one of the key components in Si photonics with extensive applications, and they can be monolithically integrated on the 3 μm SOI platform [29]. As schematically illustrated in Figure 22, the Ge PD is butt‐coupled to SOI waveguide for highly efficient light collection and detection. The Ge waveguide sidewalls are doped to ensure a highly uniform E‐field in the *i*‐region with low bias.

**FIGURE 22:**
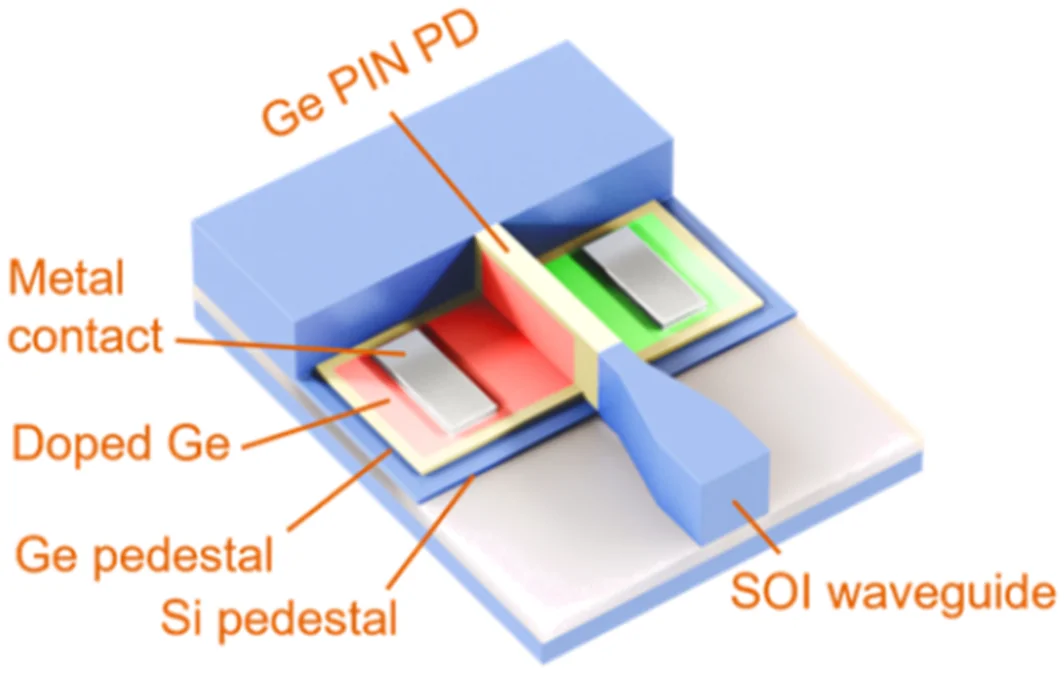
Schematic illustration of a Ge PD integrated with a SOI waveguide.

A high responsivity of ∼1 A/W has been demonstrated at 1550 nm, with a dark current of ∼5 μA when −1 V bias is applied. If the PD is used as monitor detector with smaller bias, for example, −0.1 V, the dark current can be further reduced to ∼50 nA.

Frequency responses of two typical Ge PDs are shown in Figure 23. As can be seen, a 3 dB bandwidth of ∼40 GHz has been achieved by 1 μm wide Ge PD, which has paved the way for high bandwidth applications in 3 μm SOI.

**FIGURE 23:**
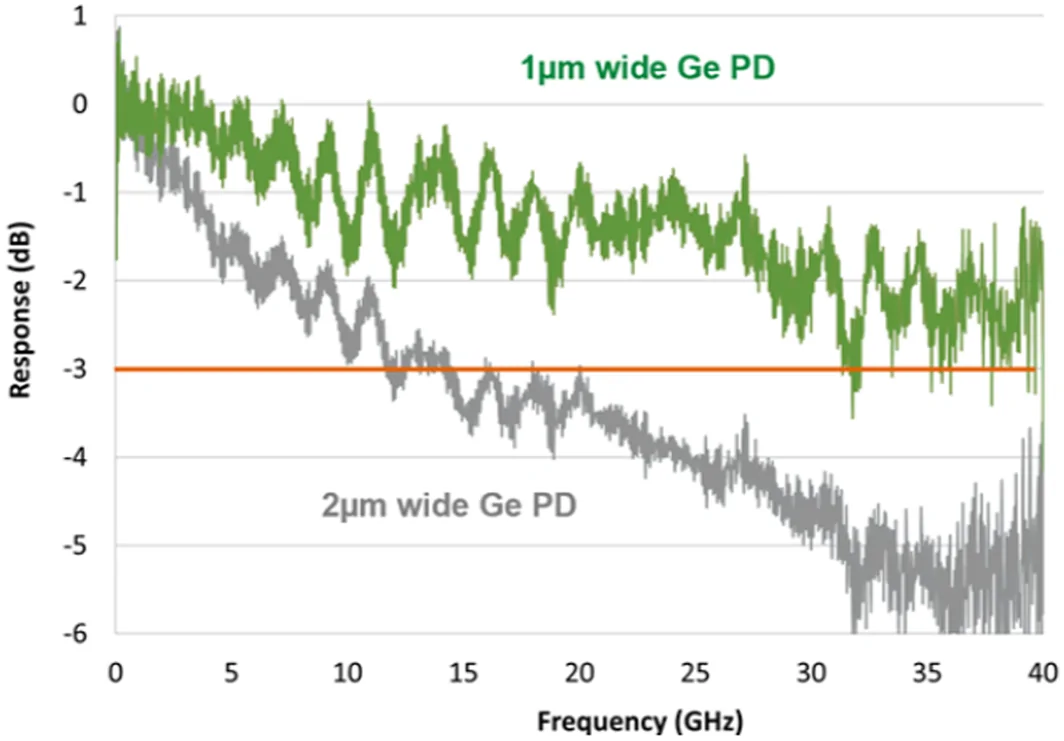
Measured frequency responses of two typical Ge PDs, showing the impact of the Ge waveguide width on bandwidth.

## 6 Hybrid Integration and Packaging

### 6.1 Flip‐Chip Bonding

Flip‐chip bonding is a critical technology for heterogeneous photonic integration, requiring precise alignment and robust bonding to achieve optimal optical performance. Here we report VTT's recent advancements in flip‐chip bonding using the Finetech Femto 2, which offers a pick‐and‐place accuracy of ± 0.3 μm. While pick‐and‐place accuracy does not guarantee post‐bonding precision, VTT has developed and optimized bonding conditions to achieve submicron post‐bonding alignment. Figure 24 presents SEM and infrared (IR) images captured following the Au‐Sn flip‐chip bonding process. While SEM imaging alone is insufficient for reliably detecting misalignment between the components, IR microscopy measurements are employed to evaluate and verify the alignment between the SOI waveguides and the III‐V compound semiconductor laser structures. This approach enables precise assessment of the bonding quality and the spatial correspondence of the integrated photonic elements. Figure 24b shows that the waveguide alignment achieved is exceptionally precise. The lateral misalignment between the III–V chip and the SOI chip remains well below 0.3 μm for both the longitudinal direction (air gap width in *x* direction) and the transverse waveguide alignment (*y*). By optimizing the flip‐chip bonding condition, a lateral alignment accuracy of approximately 0.5 μm in both the *x*‐ and *y*‐axes was achieved for more than 80% of the bonded chips. Horizontal *xy*‐alignment is made using the camera optics of the flip‐chip bonder. Linear alignment features on the chips provide *y*‐alignment, while minimizing the visible air gap between the chips enables *x*‐alignment. For vertical alignment (*z*), a mechanical stopper was designed to make contact with the III–V chip surface, enabling automatic (fully passive) adjustment of the vertical position during the bonding process. Theoretically, the vertical (*z*) alignment accuracy should be much better than ± 0.3 μm since the stopper height can be controlled with better than ± 0.1 μm accuracy. However, due to the resolution limits of IR imaging, the misalignment ranges in all three dimensions only represents preliminary estimates and cannot be measured with sufficient precision. Consequently, the alignment accuracy was indirectly validated through optical coupling measurements.

**FIGURE 24:**
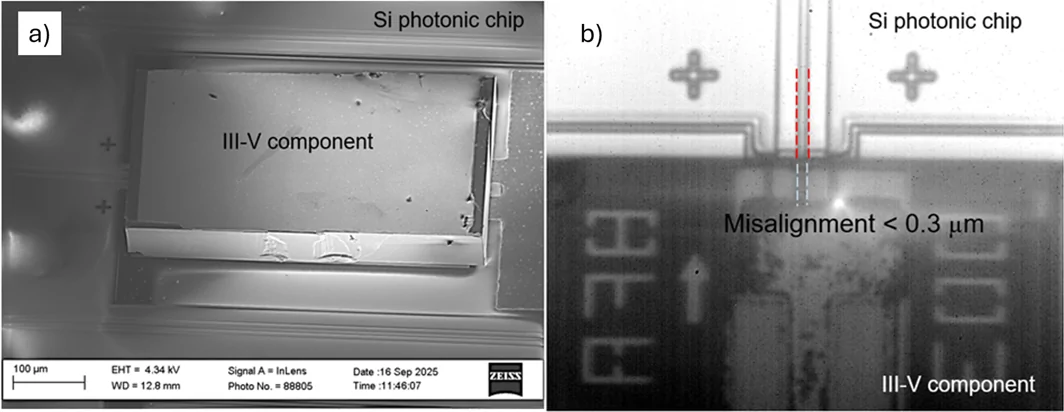
(a) SEM image of a III–V component after flip‐chip bonding on SOI. (b) IR image after flip‐chip bonding.

Figure 25 illustrates the optical output power recorded from the flip‐chip bonded device, providing a clear indication of the integrated photonic device's operational efficacy. At a drive current of 200 mA, the measured output power is 4.55 dBm, which is 1.63 dB lower than that of the standalone laser sample, which produces 6.18 dBm. This reduction in output power is predominantly attributed to mode mismatch between the components, rather than any misalignment of the laser to the waveguide. Consequently, by refining both the III–V laser chip and the silicon waveguide design, there is significant potential to further enhance the optical performance of the integrated system.

**FIGURE 25:**
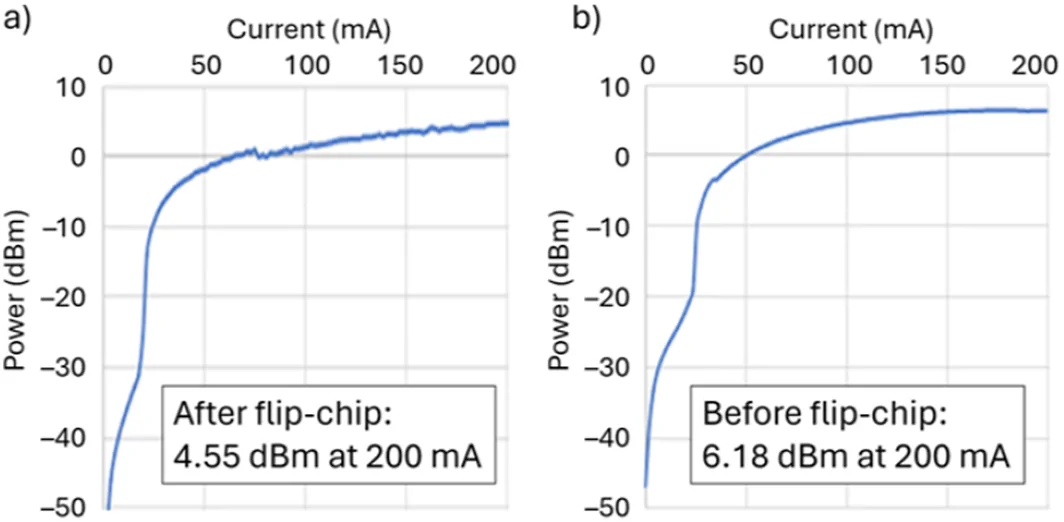
Optical power measurement (a) after flip‐chip integration and (b) for stand‐alone laser chip.

Based on the measured 1.63 dB coupling loss and our coupling loss simulations, it can be stated that the misalignment in all three dimensions is definitely below ± 1 μm and probably within ± 0.5 μm. The impact of rotational misalignment around the vertical axis is expected to be negligible as that angle can be precisely optimized and it has very little impact on coupling efficiency. Tilt of the flip‐chip bonded chip around *x* or *y* axis can have bigger impact, especially when trying to couple light in and out from the opposite ends of a long III–V chip, but that is difficult to measure and the effect would effectively translate into *xyz*‐misalignment in the ends of the III–V chip.

### 6.2 Packaging

Direct coupling from a standard single‐mode fiber (SSMF) to a 3 μm SOI waveguide typically incurs losses exceeding 5 dB due to mode size mismatch. We have demonstrated permanent low‐loss (< 2 dB) coupling using an interposer chip fabricated by mechanical polishing [30]. In the interposer, the waveguide height is adiabatically tapered from 12 to 3 μm, ensuring that the input vertical mode field matches the fiber while the output mode field aligns with the SOI waveguide.

We have also demonstrated low‐loss (1.5–2 dB) and polarization‐insensitive coupling via polymer microlenses printed directly on the waveguide facet. This approach relaxes alignment tolerances thanks to the larger mode field compared to the interposer technology. The lenses were fabricated using a two‐photon polymerization (2PP) technique [31]. An SEM image of the lenses on the waveguide facet is shown in Figure 26. The lenses were 3D‐printed with a Vanguard Sonata 1000 system. Measured spectra for edge‐coupling from a waveguide to a SSMF with and without the polymer lenses are shown in Figure 27 with 1 dB bandwidth reaching ∼685 nm.

**FIGURE 26:**
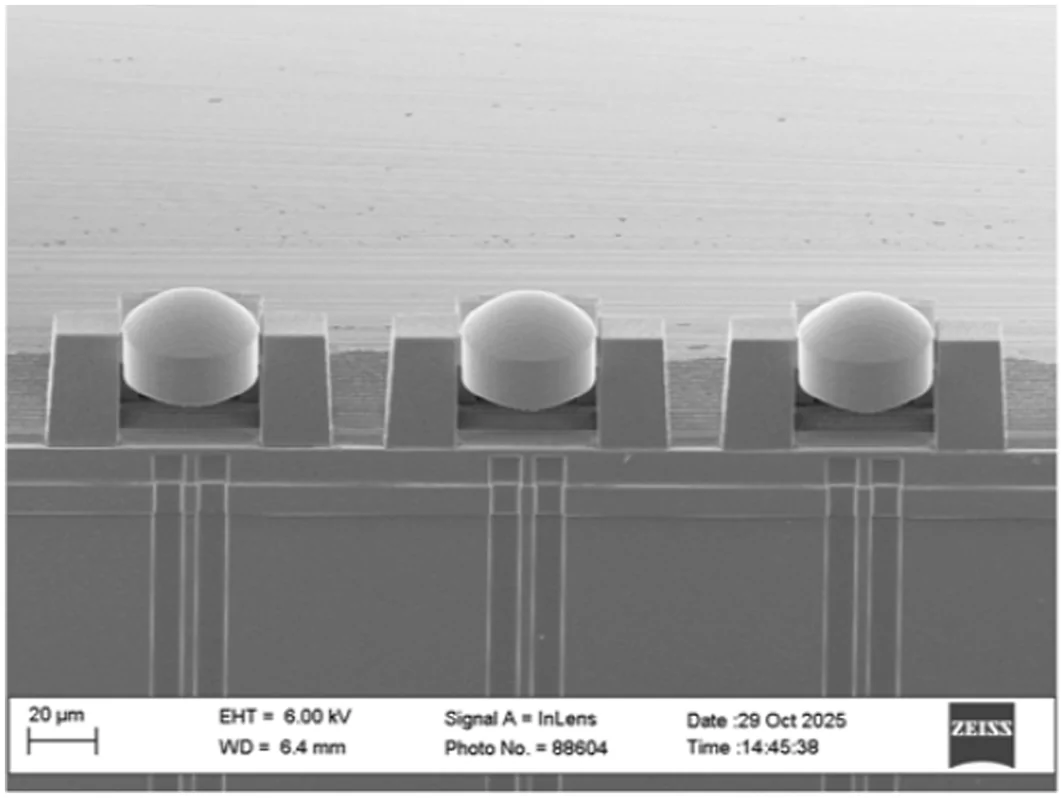
SEM image of fabricated polymer lenses on the waveguide facet for < 2 dB coupling loss to standard SM fibers.

**FIGURE 27:**
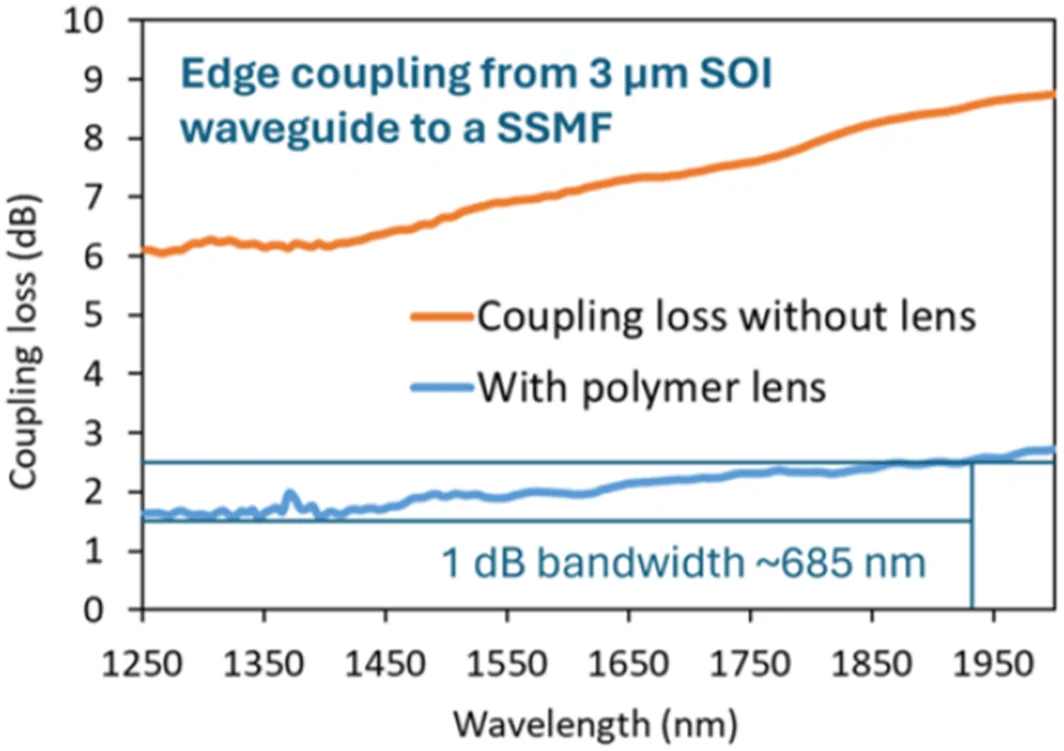
Measured fiber coupling loss for a 3 μm SOI waveguide.

## 7 Application Examples

### 7.1 Tunable External Cavity Lasers

Tunable lasers with narrow linewidth and wide tuning range can be used in many applications, such as optical communication, spectroscopic sensing, and LiDARs. Integrating external cavity lasers (ECL) from the typical shoe‐box size into chip‐scale size is a great challenge and opportunity. Widely tunable ECLs use a wavelength‐selective filter in the PIC to choose the lasing wavelength. The filter can be realized in several ways, typically using ring resonators in Vernier configuration [32] or cascaded MZIs [33]. Other configurations such as reflection‐type transversal filters have also been demonstrated [34]. Optically passive PIC platforms such as SOI and Si_3_N_4_ require the optical gain material to be either hybrid‐ or heterogeneously integrated with the PIC. This is typically done by end‐fire coupling an III–V reflective semiconductor optical amplifier (RSOA) chip to the PIC chip. For hybrid integration on the 3 μm SOI platform, the gain chips can be flip‐chip mounted, providing a robust and compact ECL solution.

We have recently demonstrated a tunable laser in a three‐ring resonator tunable reflector in 3 μm SOI based on the Vernier effect [35] in the DYNAMOS EU project. An InP RSOA was fiber‐coupled to the SOI chip as the light source. A tuning range of ∼60 nm was achieved with mW‐level output power and a side‐mode suppression ratio of ∼45 dB (Figure 28). The design utilizes microheaters as phase shifters for ∼30 kHz tuning; future design iterations will focus on integrating carrier injection phase shifters for faster tuning.

**FIGURE 28:**
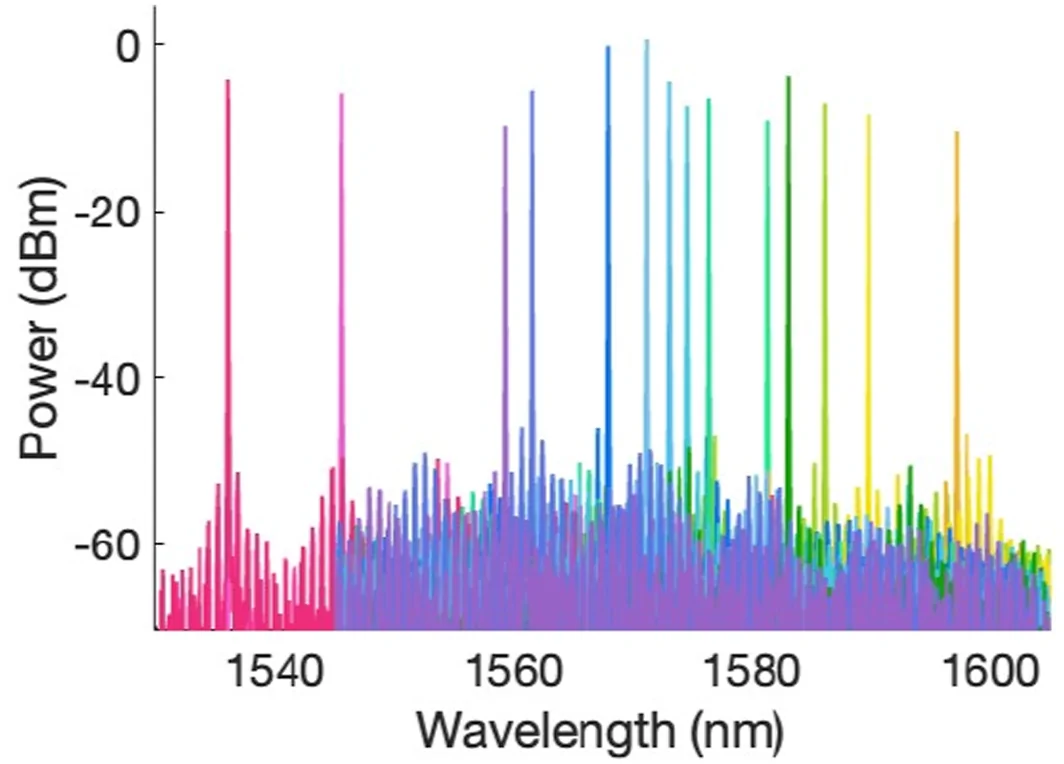
Measured output spectra with different heating powers to show the tunability of an external cavity laser.

### 7.2 Integrated Photonic Ultrasound Transducers

Ultrasound sensing is a widely used medical imaging modality that enables real‐time, non‐ionizing, and comparatively low‐cost diagnostics [36]. Current clinical systems rely mainly on piezoelectric transducers but emerge of capacitive‐based and piezoelectric‐based micromachined ultrasound transducers (CMUT and PMUT) have overcome many performance limitations given by bulk transducers, specifically in terms of frequency of operation and acoustic impedance matching to biological tissues and ease in fabricating electrical interconnections to highly dense transducer arrays [37].

Optical and photonic ultrasound sensors are a strong alternative for medical applications. In optical ultrasound sensing, acoustic waves interact with optical fields and create pressure‐induced changes in refractive index, optical phase, or optical cavity length, which can be measured using interferometric or resonant optical structures. All‐optical detectors yield a higher signal‐to‐noise ratio per unit area and feature wider detection bandwidths, which is preferable especially for optoacoustic applications, and are less sensitive to electromagnetic fields [38]. Technical approaches include Fabry–Pérot sensors [39], fiber Bragg gratings [40, 41], and different PIC‐based approaches, for example, ring resonators [42], Mach–Zehnder interferometers [43], and Bragg gratings [44, 45]. State‐of‐the‐art devices have demonstrated bandwidths extending into the tens of megahertz and, in optimized Fabry–Pérot sensors, showing potential beyond 100 MHz [46]; meanwhile optomechanical microcavity devices have reached noise‐equivalent pressures in the nPa/√Hz range under laboratory conditions [47].

In EU project Med‐IPUT [48], in collaboration with TNO, we have developed integrated photonic ultrasound sensors (IPUT) on VTT's 3 μm SOI platform. The IPUT sensor is an interferometric sensor where a long spiral waveguide placed on a mechanical membrane structure forms the sensing arm of an MZI, and a waveguide spiral without the membrane forms the reference arm of the MZI. When the sensor is exposed to ultrasound, the pressure wave causes the membrane to vibrate. A time‐periodic strain in the spiral waveguide modulates the waveguide length and effective index. The induced phase modulation of the guided wave is detected as an intensity modulation at the output of the MZI.

By optimizing the waveguide spiral design and the back‐side etching process of the membrane, we have fabricated cascaded IPUT devices (Figure 29) which show a significant improvement in the measurable noise equivalent pressure (NEP) of the sensor [49]: NEP values of 35× lower than in conventional state‐of‐the‐art transducers have been demonstrated. This paves the way for more sensitive ultrasound receivers that could enable, for example, higher image quality and deeper imaging depth in future medical ultrasound imaging.

**FIGURE 29:**
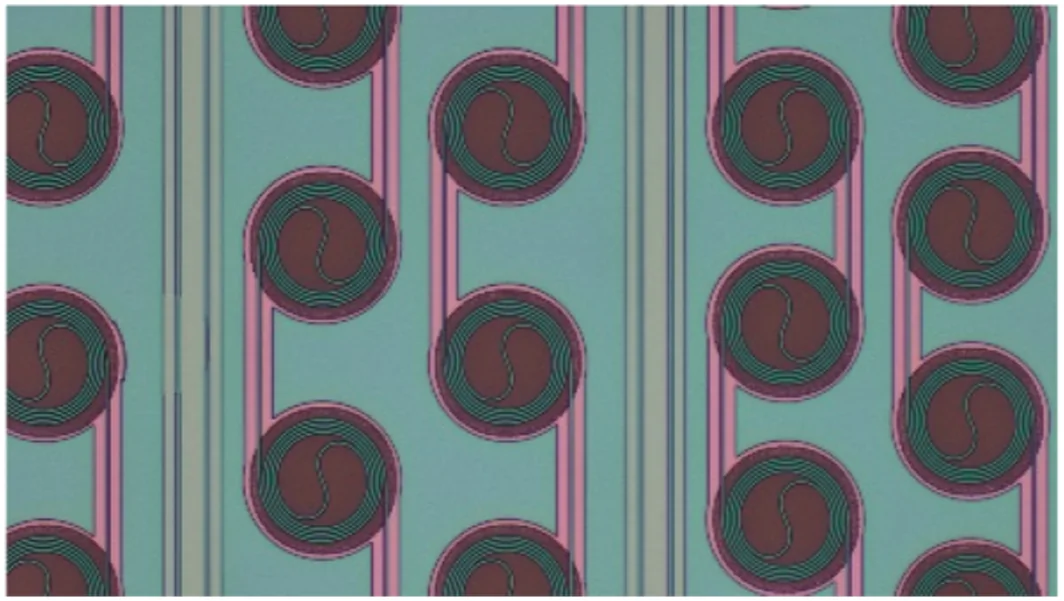
A microscope image of cascaded waveguide spirals on membranes fabricated using VTT's 3 μm SOI platform. The membrane diameter is about 110 μm.

### 7.3 Optical Phased Arrays for Beam Steering

Small integrated LiDAR has many application areas like automotive [50], drones [51], and robotics [52]. OPA offers a solid‐state beam steering solution that is light weight and immune to vibration. There are also other applications for chip scale OPAs, such as free space communication [53] and imaging [54]. Active phase shifters in OPAs has been shown even with 1024 channels [55]. Two‐dimensional beam steering has also been presented combining 16 channel OPA with active phase shifters to waveguide gratings [56].

We have developed passive OPAs for LiDAR laser beam steering in the 1300 and 1550 nm range [57, 58]. Our OPA design has two different versions with 2 and 3 μm waveguide spacing *d* (Figure 30). Both devices have 512 waveguides and the same 14.5 μm constant physical length difference between adjacent waveguides. Phase difference between waveguides can be tuned passively with wavelength tuning. Active phase tuning elements are not needed due to the small phase errors in 3 μm SOI waveguides. Our passive OPA is much simpler and less power‐consuming with large channel numbers than an active OPA controlled by phase shifters. Both 2 and 3 μm waveguide spacing devices have the same 5 × 10 mm^2^ footprint. A star coupler provides a Gaussian envelope profile for the intensity distribution of the waveguide array which eliminates side slopes in the output. The waveguide arrays in the output are 1 and 1.5 mm wide, corresponding with the 2 and 3 μm waveguide spacing in the array. The maximum beam steering angles with 45 nm wavelength tuning are 45° and 30° for 2 and 3 μm devices, respectively. The steering sensitivities are about 1°/nm and 0.6°/nm at 1550 nm for the 2 and 3 μm devices, respectively.

**FIGURE 30:**
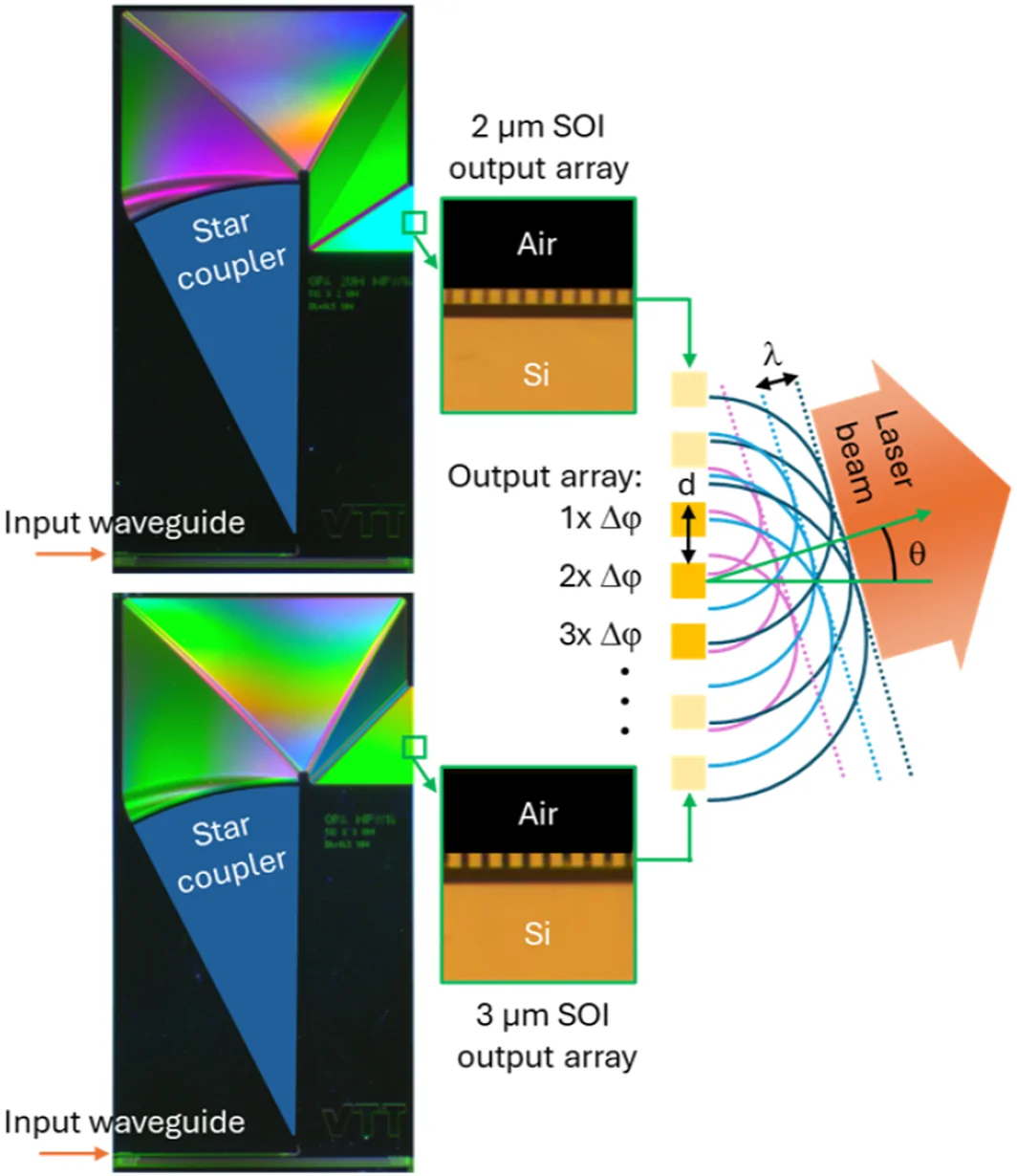
Microscope images of OPA chips with 2 μm (upper) and 3 μm (lower) output waveguide pitch (left). The output array for 1D beam steering is shown in the middle. Phase difference Δ*φ* induced by wavelength tuning defines the steering angle *θ*, as schematically illustrated on the right.

While the waveguide array collimates the output beam in the horizontal direction, a lens is needed for vertical collimation. A cylindrical lens is used to collimate the beam in the vertical direction; however, due to the large beam steering angle, projected lens cross‐section and focal distance are not constant when straight cylindrical lens is used. We have thus developed a 3D‐printed curved cylindrical lens that can collimate output light for large steering angles [58].

We tested LiDAR operation with a frequency modulated continuous wave (FMCW) distance measurement [57] using the OPA and a tunable laser (Santec, TSL‐550) with 100 nm/s tuning speed. Frequency difference between the reference and signal arms was measured with a balanced detector (Thorlabs, F230FC‐155) using fast Fourier transform (FFT) with an oscilloscope (Tektronics, TDS3014). Distances of 41–336 cm were measured with a linear frequency response of 1 cm/kHz to an accuracy of ∼5 cm.

### 7.4 Optical True Time Delays for RF Beam Steering

Microwave phased array antennas play a key role in dynamic bandwidth allocation for increased wireless communication demand, as they enable precise electronic beam steering for radar, satellite communication, and next‐generation wireless applications [59, 60]. In contrast to purely electronic approaches, OTTD solutions deliver much higher bandwidth, strong immunity to electromagnetic interference, and the benefits of compact photonic integration [61]. Integrated optical delay lines offer significant improvements in size, scalability, power efficiency, and overall cost [62]. In recent years, different schemes such as ring resonator [63], MZI switch [64], photonic crystal [65], and waveguide Bragg grating [66] based integrated optical delay lines have been presented on various material platforms [67].

We developed a 5‐bit, 4‐channel reconfigurable OTTD chip integrated on VTT's 3‐μm SOI platform. A block diagram of the 5‐bit reconfigurable OTTD system is shown in Figure 31. Thermo‐optic phase shifter‐based MZI switches are used between the delay stages. Each channel can deliver a tunable delay from 0 to 100 ps in 32 discrete steps, enabling full 0–2π phase control for a 10 GHz RF carrier.

**FIGURE 31:**
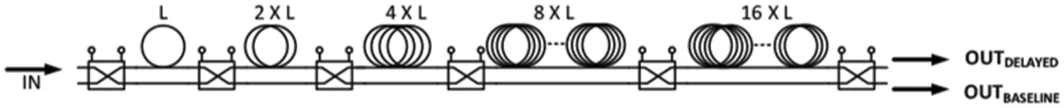
Block diagram of a 5‐bit reconfigurable OTTD.

The OTTD chip was incorporated into a radio‐over‐fiber architecture and connected to a four‐element RF antenna array. Free‐space RF beam steering was experimentally demonstrated, with performance validated between broadside and 30° steering angles. Pictures of the optically and electrically packaged assembly and the diced OTTD chip are shown in Figure 32.

**FIGURE 32:**
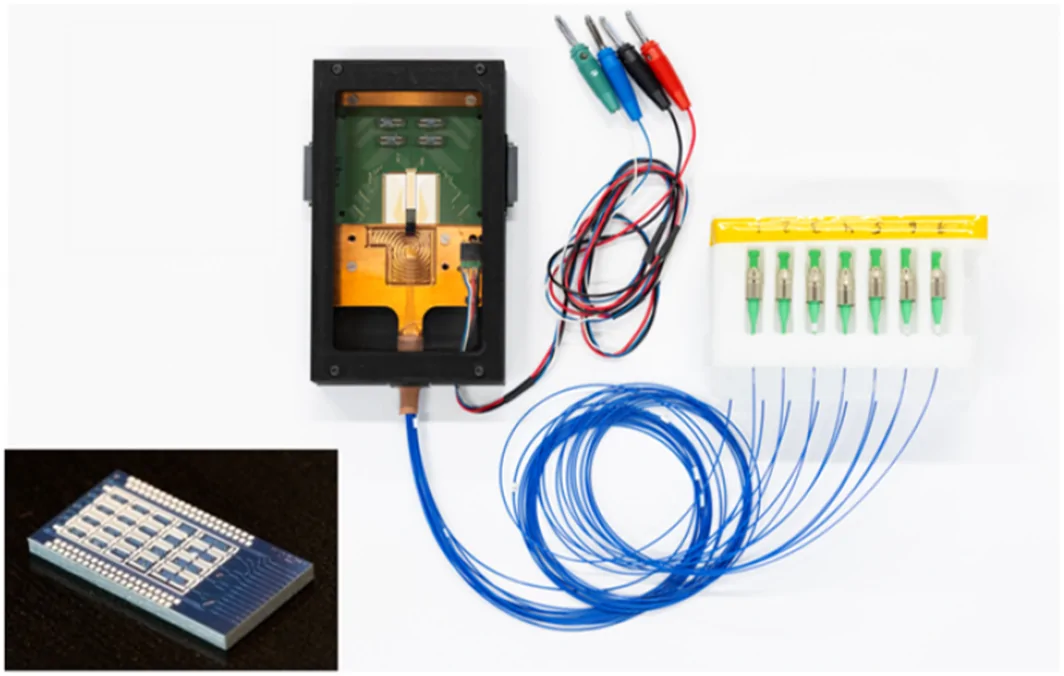
Picture of the assembly with fiber array and electrical packaging. Picture of the diced OTTD chip is in the inset.

The chip was calibrated, and a lookup table was generated to introduce the intended time delay for each channel to form the final RF beam in the desired direction. The performance of the packaged OTTD chip was evaluated using a 4‐channel high‐speed oscilloscope, as shown in Figure 33. Two distinct beamforming directions were studied: broadside and 30° steering.

**FIGURE 33:**
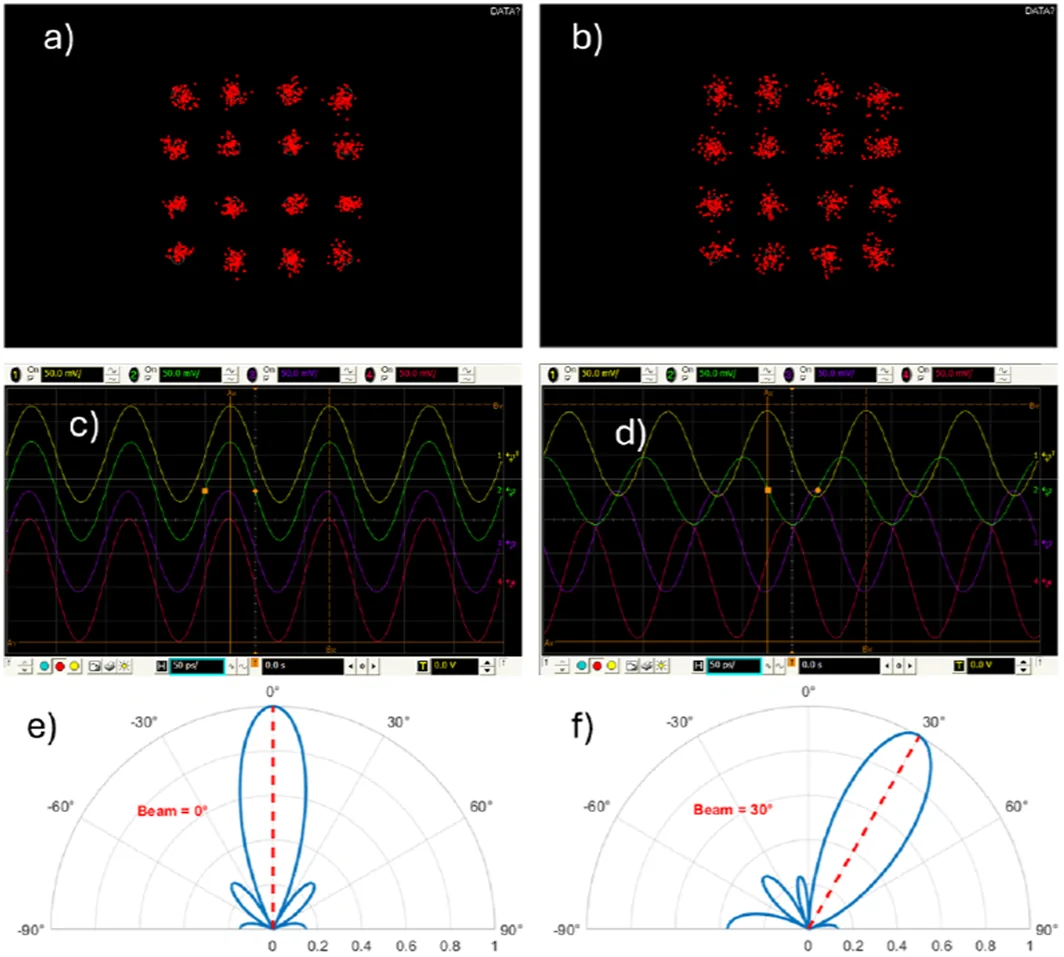
Constellations of 16‐QAM 100 MBaud baseband data on 10 GHz carrier after free space propagation for (a) broadside beam with error vector magnitude (EVM) 8%, and (b) steered beam at 30° with EVM 9%. (c, d) Show the oscilloscope waveforms for 10 GHz carrier signal with 0 ps relative time delay between the channels for broadside beam, and 25 ps relative time delay for beam formed at 30°, respectively. (e, f) Show the RF beam direction and shape calculated with the array factor for broadside and 30° beam, respectively.

For the given RF transmitter antenna geometry, the required relative inter‐element time delay for 30° beam steering was 25 ps. Accordingly, a progressive delay of 25 ps was applied between the four channels. The exact delays programmed into the four channels were 0, 25, 50, and 75 ps, respectively. An antenna receiver was placed 5 m away from the transmitter at a 30° angle to capture the radiated waveform. The constellation diagrams of the transmitted data for both broadside and 30° beam steering cases are shown in Figure 33.

## 8 Comparison to Other PIC Platforms

The most common PIC technologies are now based on silicon, silicon nitride, silicon dioxide, indium phosphide, lithium niobate, and polymers (as the primary core material); meanwhile, emerging and less mature PIC platforms include, for example, barium titanate, silicon carbide, germanium, gallium arsenide, and aluminum oxide or titanium oxide. All these have their own unique benefits and limitations. In the literature, there are also many reported combinations of different PIC platforms using heterogeneous or hybrid integration, which makes the detailed and quantitative comparison to all possible PIC platforms very difficult. Figure 34 shows a qualitative comparison of our 3 μm SOI platform with some of the most commonly used monolithic PIC platforms and with respect to some of the basic PIC functionalities. This shows how each PIC platform offers some unique benefits in photonic functionalities, technology scalability and maturity, and/or wavelength range. The main benefits of the 3 μm SOI PIC technology are low losses, dual‐polarization operation, and the ultra‐broadband operation from SWIR to MIR wavelengths.

**FIGURE 34:**
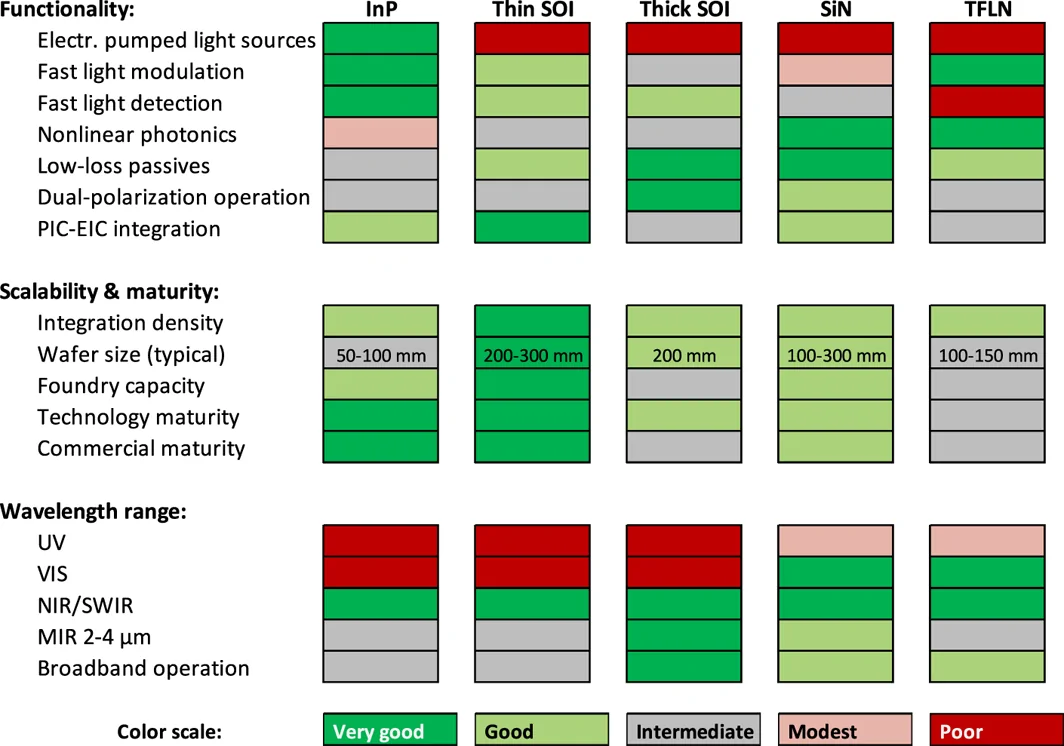
Qualitative comparison of 3 μm SOI with some of the most common other monolithic PIC platforms. (MIR = mid infrared, NIR = near infrared, SWIR = short wave infrared, UV = ultraviolet, VIS = visible).

In Table 1, we make a more detailed and quantitative comparison to 220 nm SOI PICs. The main strengths of 3 μm SOI PIC technology are lower losses, tolerance to higher optical powers, dual‐polarization operation, and ultra‐broadband operation. The main deficiencies relate to the lack of fast modulators and the mature manufacturing ecosystem.

**TABLE 1:** Quantitative comparison of 3 μm SOI with the mainstream 220 nm SOI PIC technology.

Metric	220 nm SOI	3 μm SOI	Notes
Propagation loss for typical waveguide	1–3 dB/cm for SM waveguides in most publications	0.03–0.1 dB/cm for hydrogen‐annealed and 0.1–0.2 dB/cm for thermally oxidized rib and strip waveguides	Lower loss in thick SOI due to small mode overlap with sidewall roughness
Propagation loss, best demonstrated	0.065 dB/cm in a race track resonator with 2–3 μm wide MM strip waveguides, reaching intrinsic *Q* value up to 10 million [68]	0.027 dB/cm in a race track resonator with a combination of rib and strip waveguides, reaching intrinsic *Q* value up to 14 million [1]	Best‐case values converge, but thick SOI achieves them in standard routing waveguides
Minimum bend radius (*R*) and bend loss	Typical loss < 0.01 dB/90° at *R* ≥ 5 μm or larger; optimized L‐ and U‐bends demonstrated with 2.5 μm × 2.5 µm and 3.0 μm × 3.6 μm footprint and 0.07 dB loss [69]	Typical loss < 0.02 dB/90° at *R* ≥ 10 μm with Euler bends in strip waveguides; smallest bends demonstrated with < 0.1 dB/90° loss for both polarizations using 1 μm minimum *R* [12]; TIR mirrors with 0.1–0.2 dB loss.	Both achieve μm‐scale bends. Dense routing in thick SOI requires either conversion to a strip waveguide or TIR mirrors, since thick rib waveguides require mm‐scale radii
Integration density	Sub‐µm waveguides; ring modulators with ∼10–20 μm diameter; on‐chip data‐rate density 1.6 Tb/s/mm^2^ demonstrated [70]	Bends and mirrors with μm‐scale *R*; waveguide pitch 2–3 μm; cm‐long spirals in < 1 mm^2^ footprint; waveguide crossings with ∼0.02 dB IL without tapering	220 nm wins for ultimate density, but 3 μm SOI is far denser than other micron‐scale platforms.
Optical power limitation from TPA, FCA, and other nonlinear processes at telecom wavelengths	Maximum power per wavelength is typically limited to 3.2 mW due to unwanted nonlinearities in ring resonators. Maximum total power per waveguide is typically limited to the 30–100 mW range. As a function of input power, the absorption first increases and the total output power eventually saturates [71].	Propagation loss starts to increase at ∼1 W total power per waveguide.	Maximum optical power is tens of times higher in thick SOI due to larger mode field, which is beneficial in, for example, scaling up the number of wavelength division multiplexing (WDM) channels or the power of LiDAR systems.
Polarization dependence	Strong form birefringence and substantial polarization‐dependent losses; standard grating couplers are single‐polarization; polarization‐diversity circuits typically required.	Down‐to‐zero birefringence; polarization‐dependent loss in components and fiber coupling is typically very small; residual strain birefringence is trimmed via waveguide width.	Major differentiator: Thick SOI is natively polarization‐insensitive
Coupling loss to a standard SM fiber (SSMF)	Grating couplers typically have 3–6 dB loss with 30–50 nm of 1 dB bandwidth. Best results with more complicated fabrication have < 2 dB loss. These typically operate at single polarization. Typical edge couplers provide 1.5–2 dB coupling loss to small‐core fibers (not SSMF) with both polarizations [72]. Lowest demonstrated coupling loss to SSMF is 0.8 dB (3 dB) with a 1 dB bandwidth > 180 nm (130 nm) for TE (TM) using a printed 3D lens [73].	Edge‐coupling loss ∼0.5 dB to lensed/small‐core fiber (MFD ≈ 2.5 μm) and ∼1.5 dB to SSMF with 1 dB bandwidth ∼685 nm; vertical up‐reflecting mirrors with similar losses; all fiber coupling solutions are essentially polarization independent.	Thick‐SOI mode size is closer to fiber and fiber coupling losses are in general lower with the added benefit of having polarization independent and ultra‐broadband coupling.
Fastest modulators in SOI without hybrid or heterogeneous integration	Carrier injection modulators reach 1–20 GHz, while carrier‐depletion modulators reach up to 110 GHz bandwidth [74]. Modulators are often integrated into micro ring resonators to combine modulation with wavelength multiplexing.	Carrier injection modulators reach 10–50 MHz bandwidth; 15 Gbps NRZ modulation was demonstrated in a 1.8 μm thick‐SOI waveguide with 24 V DC reverse bias and 9.5 V peak‐to‐peak voltage [75].	Lack of monolithic high‐speed (> 10 GHz) modulators is the most significant weakness of the 3 μm SOI PIC platform.
Monolithic Ge photodetectors	Typical bandwidth 40−100 GHz and responsivity 0.7−0.1 A/W at ∼1 V reverse voltage; 265 GHz with 0.3 A/W at 2 V reverse bias is the fastest demonstration [76].	> 40 GHz bandwidth at 1 V reverse bias and ∼1 A/W responsivity demonstrated [29].	Both support 56+ Gbaud detection; 220 nm SOI offers somewhat faster typical performance and much better bandwidth record.
Wavelength range of operation	Single‐mode operation with low bending losses can be achieved over ∼300 nm bandwidth; cladding absorption makes operation impractical beyond ∼2.5 µm	Effectively single‐mode PIC operation with low bending losses can be achieved over ∼3 μm bandwidth; rib design can be single‐moded from 1.2 to > 4 μm; high confinement enables low‐loss operation up to 3–4 μm wavelength;	Thick SOI offers uniquely broadband operation
Wafer size and scalability to production	Typically 200–300 mm wafers; excellent scalability to mass production in CMOS foundries	Typically 200 mm wafers; possibility to scale up to mass production in a few foundries	220 nm SOI has far larger manufacturing ecosystem

## 9 Conclusions and Outlook

We herein present an overview of the 3 μm SOI PIC platform and explain its unique characteristics, such a ultra‐high mode confinement, low losses, possibility for dense integration, polarization independent operation, small phase errors, ultra‐broadband operation, and high optical power handling up to ∼1 W. Some examples of monolithically integrated passive and active waveguide components were given, including couplers, wavelength (de)multiplexers, phase modulators, and Ge photodiodes. Also, hybrid integration and packaging possibilities were briefly addressed. The feasibility of the 3 μm SOI PIC platform was demonstrated for several example applications: tunable lasers, optical beam steering (e.g., FMCW LiDAR), medical imaging, and RF beam steering.

Ongoing and targeted developments for 3 μm SOI include layer transfer for multilayer PICs, adoption of deep‐ultraviolet lithography for improved patterning resolution, and the heterogeneous integration of other materials at the chip‐ and wafer‐level for complementary functions, such as light sources, fast modulation, and single‐photon detection. Open access to this PIC platform will be expanded with the PIXEurope pilot line [6], where new PIC functionalities are developed, and process design kits (PDKs) are published. The process line was upgraded from 150 to 200 mm wafer size in August 2025.

## Author Contributions

**Timo Aalto:** conceptualization, investigation, funding acquisition, writing – original draft, methodology, visualization, writing – review and editing, supervision, resources. **Dura Shahwar:** investigation, writing – original draft, visualization, methodology, validation. **Markku Kapulainen:** investigation, writing – original draft, methodology, validation. **Mikko Harjanne:** investigation, writing – original draft, methodology, validation, visualization, software. **Katherine Bryant:** investigation, writing – original draft, methodology, validation. **Somnath Paul:** investigation, writing – original draft, methodology, validation, visualization. **Päivi Heimala:** investigation, writing – original draft, methodology, funding acquisition, project administration, resources. **Fei Sun:** investigation, writing – original draft, validation, methodology. **Jae‐Wung Lee:** investigation, writing – original draft, methodology, validation. **Ari Hokkanen:** investigation, writing – original draft, methodology, validation, visualization.

## Funding

This work has been partially funded by (1) the European Commission (doi: 10.13039/501100000780) in projects DYNAMOS (Grant 101070342, https://www.project‐dynamos.eu/), Med‐IPUT (Grant 101092947, https://med‐iput.eu/) and PIXEurope (Grants 101213727 and 101213744, https://pixeurope.eu/), (2) Business Finland (doi: 10.13039/501100014438) in projects PIXEurope (Grant 5823/31/2024) and PICAP (Grant 44065/31/2020), and (3) the Research Council of Finland (doi: 10.13039/501100002341) in project NOIMO (Grant 333982), in the photonics flagship programme PREIN (Grants 346545 and 368651, https://prein.fi/), and in the Finnish national infrastructure for light‐based technologies (Grant 359148, https://finnlight.fi/).

## Conflicts of Interest

The authors declare no conflicts of interest.

## Data Availability

The datasets generated and/or analyzed during the current study are available from the corresponding author upon reasonable request.
